# Genetic diversity and regulatory features of human-specific *NOTCH2NL* duplications

**DOI:** 10.1101/2025.03.14.643395

**Published:** 2025-03-17

**Authors:** Taylor D. Real, Prajna Hebbar, DongAhn Yoo, Francesca Antonacci, Ivana Pačar, Mark Diekhans, Gregory J. Mikol, Oyeronke G. Popoola, Benjamin J. Mallory, Mitchell R. Vollger, Philip C. Dishuck, Xavi Guitart, Allison N. Rozanski, Katherine M. Munson, Kendra Hoekzema, Jane E. Ranchalis, Shane J. Neph, Adriana E. Sedeño-Cortes, Benedict Paten, Sofie R. Salama, Andrew B. Stergachis, Evan E. Eichler

**Affiliations:** 1 Department of Genome Sciences, University of Washington School of Medicine, Seattle, WA 98195, USA; 2 Department of Biomolecular Engineering, University of California, Santa Cruz, Santa Cruz, CA 95064, USA; 3 UC Santa Cruz Genomics Institute, University of California, Santa Cruz, Santa Cruz, CA 95060, USA; 4 Department of Biosciences, Biotechnology and Environment, University of Bari, Bari, 70125, Italy; 5 College of Natural & Agricultural Sciences, University of California, Riverside, Riverside, CA 92521, USA; 6 Department of Psychology and Neuroscience, University of North Carolina, Chapel Hill, Chapel Hill, NC 27514, USA; 7 Division of Medical Genetics, Department of Medicine, University of Washington School of Medicine, Seattle, WA 98195, USA; 8 Department of Molecular, Cell and Developmental Biology, University of California, Santa Cruz, Santa Cruz, CA 95064, USA; 9 Brotman Baty Institute for Precision Medicine, Seattle, WA 98195, USA; 10 Howard Hughes Medical Institute, University of Washington, Seattle, WA 98195, USA

**Keywords:** Segmental duplication, human evolution, gene duplications, *NOTCH2*, *NOTCH2NL*

## Abstract

*NOTCH2NL* (*NOTCH2*-N-terminus-like) genes arose from incomplete, recent chromosome 1 segmental duplications implicated in human brain cortical expansion. Genetic characterization of these loci and their regulation is complicated by the fact they are embedded in large, nearly identical duplications that predispose to recurrent microdeletion syndromes. Using nearly complete long-read assemblies generated from 67 human and 12 ape haploid genomes, we show independent recurrent duplication among apes with functional copies emerging in humans ~2.1 million years ago. We distinguish *NOTCH2NL* paralogs present in every human haplotype (*NOTCH2NLA*) from copy number variable ones. We also characterize large-scale structural variation, including gene conversion, for 28% of haplotypes leading to a previously undescribed paralog, *NOTCH2tv.* Finally, we apply Fiber-seq and long-read transcript sequencing to human cortical neurospheres to characterize the regulatory landscape and find that the most fixed paralogs, *NOTCH2* and *NOTCH2NLA*, harbor the greatest number of paralog-specific elements potentially driving their regulation.

## INTRODUCTION

Notch signaling, a mechanism of cell communication conserved throughout the metazoan kingdom, is uniquely altered in humans due to a recent ape segmental duplication of the *NOTCH2* gene (Fiddes et al. 2018, Suzuki et al. 2018). Segmental duplications (SDs) have both restructured primate genomes as well as led to the emergence of lineage-specific gene families resulting in potentially new functions, including distinct developmental fates (Yoo et al. 2025, Florio et al. 2018). In the case of *NOTCH2NL* (*NOTCH2-N-terminus-like*), the gene family is a ~70 kbp SD encompassing the four N-terminal exons of *NOTCH2* and includes a unique final fifth exon exapted from the fourth intron of *NOTCH2*. While *NOTCH2NL*-like sequences exist in several primates, the NOTCH2NL protein appears to only be expressed in the developing human brain, suggesting it is functionally human specific (Fiddes et al. 2018). Experimental work has shown that NOTCH2NL interacts with NOTCH2 and modulates the NOTCH2-signaling pathway (Fiddes et al. 2018, Suzuki et al. 2018). Specifically, NOTCH2NL increases the number of self-renewal divisions of progenitor radial glia while delaying differentiation of these cells into neurons. Prioritizing self-renewal over differentiation has been proposed to enable the human brain to increase neuronal mass during cortical neurogenesis (Fiddes et al. 2018, Suzuki et al. 2018). The functional impact of NOTCH2NL may be one explanation for the expansion of the human ancestral brain compared to our closest living relatives, the chimpanzees.

In addition to its potential role in human brain evolution, mutations in the *NOTCH2/NL* gene family or their associated SDs underlie four distinct genetic disorders. Alagille syndrome is caused by mutations in the ancestral gene *NOTCH2* (Rajagopalan et al. 2021, Li et al. 2022). Neuronal intranuclear inclusion disease (NIID) results from a CCG repeat expansion in the 5’ untranslated region of the human-specific paralog *NOTCH2NLC* (Ishiura et al. 2019, Sone et al. 2019). Finally, the chromosome 1q21.1 distal duplication/deletion and TAR (thrombocytopenia-absent radius) syndromes are recurrent rearrangements associated with developmental disorders for which the breakpoints have been mapped to the SD regions containing the *NOTCH2NLA*, *NOTCH2NLB*, and *NOTCH2NLC* copies (Klopocki et al. 2007, Brunetti-Pierri et al. 2008, Mefford et al. 2008). Thus, there has been a mutational burden and disease predisposition associated with the emergence of these human-specific paralogs.

Historically, complex high-identity regions enriched in SDs, like the *NOTCH2NL* locus from chromosome 1p12–1q21, have been difficult to sequence and assemble with short-read technologies. Duplicated *NOTCH2NL* copies are embedded in much larger blocks (often hundreds of kbp to a few Mbp in length) of SDs associated with other genes such as the core duplicon *NBPF* (neuroblastoma breakpoint family) (Vandepoele et al. 2005, Jiang et al. 2007, Dumas and Sikela 2009, O’Bleness et al. 2012, Fiddes et al. 2018, Fiddes et al. 2019). *NOTCH2NL* gene family annotations are frequently incorrect in previous reference genome builds because of assembly gaps and collapses that can now be resolved with long-read sequencing platforms (Vollger et al. 2020, Nurk et al. 2021, Vollger et al. 2022). Additionally, because *NOTCH2NL* paralogs share >99% sequence identity, even when the region is fully assembled, they remain difficult to distinguish from one another. Mutational processes such as interlocus gene conversion (IGC) (Chen et al. 2007) make paralogous sequence variants unreliable as tags unless such IGC patterns in diverse humans are fully characterized. As a result, studies of human genetic variation have frequently excluded these regions; standard genome-wide association studies and attempts to functionally characterize via ENCODE and GTEx are almost nonexistent due to their dependence on short-read sequencing platforms (The ENCODE Project Consortium 2012, GTEx Consortium 2017).

In this study, we address these limitations by using long-read sequencing data and associated pangenome and telomere-to-telomere (T2T) resources generated from nonhuman primates (NHP) and a diverse set of humans (Human Pangenome Reference Consortium [HPRC]) (Mao et al. 2024, Yoo et al. 2025, Liao et al. 2023). We comprehensively characterize the copy number, structure, and functional features of this gene family and place these changes into the context of human-specific changes that have reshaped chromosome 1. Using comparative data from other great apes, we construct an evolutionary model for its origin and spread over the last 10 million years, providing evidence of recurrent duplication in different apes while showing that functional copies began to emerge and spread ~2 million years ago (MYA) in the human lineage. We identify *NOTCH2tv,* a novel paralog that emerged due to a more recent ancestral gene conversion event with *NOTCH2* in 10% of human haplotypes (a subset of the total IGC observed in human haplotypes). Finally, we apply Fiber-seq and long-read transcript sequencing to a cortical neurosphere model, providing an initial assessment of potential regulatory regions that are shared and those that distinguish paralogs specifically during human neurodevelopment.

## RESULTS

### Structure of the *NOTCH2NL* gene family in a complete human genome assembly

**Figure 1.**
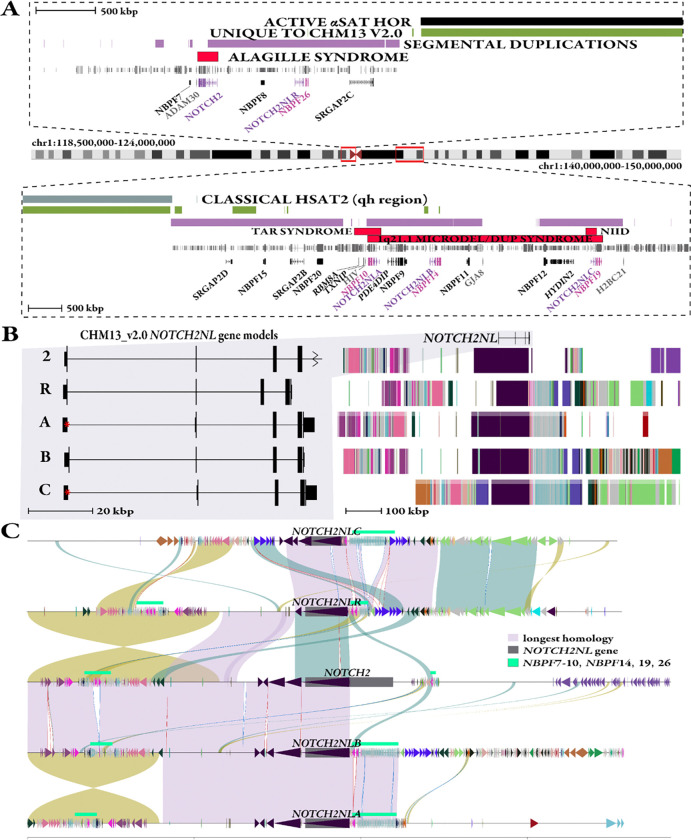
Genome structure and organization of the *NOTCH2NL* gene family. **a)** Long-range organization of *NOTCH2/NOTCH2NL* loci in T2T-CHM13 reference genome, including centromere satellite annotations of active alpha satellite (*α*Sat) higher order repeats (HORs) (black) and classical human satellite 2 (hsat2, secondary constriction [qh] region [Patil and Lubs 1977]) (blue), regions unique to the T2T-CHM13 assembly (green), intervals of SDs (purple), and Mendelian and genomic disorders associated with specific regions/paralogs (red). A subset of genes is depicted, including *NOTCH2NL* (purple), *NBPF* genes that are directly downstream of *NOTCH2NL* (pink), first unique genes located outside of SD blocks (gray), and all others (black). **b)** Duplicon organization as defined by DupMasker (Methods) flanking the *NOTCH2NL* region and intron/exon structure of genes in T2T-CHM13 V2.0 (Perez et al. 2025, http://genome.ucsc.edu). Red asterisks mark the nontraditional CTG start that the browser annotations do not take into consideration. **c)** Stacked SVbyEye plot of 1 Mbp regions flanking human *NOTCH2NL* genes (gray squares), contrasting syntenic regions in direct orientation (blue/lavender) versus inverted alignments (yellow). Annotations include different *NBPF* genes in the region (teal). Note: the two large inversions between *NOTCH2/NOTCH2NLR* and *NOTCH2NLA*/*NOTCH2NLB*, respectively, are the result of proximity due to overlapping sequence. Duplicons as defined by DupMasker (colored triangles).

To establish a framework for studying the *NOTCH2NL* gene family in humans, we first characterized the structure of this gene family along the fully assembled T2T-CHM13 haploid genome (Nurk et al. 2021). In T2T-CHM13, *NOTCH2* and *NOTCH2NLR* are clustered on the p-arm of chromosome 1 within 1.5 Mbp of the centromere (Figure 1A). *NOTCH2NLA*, *NOTCH2NLB*, and *NOTCH2NLC* map to the q-arm (Figure 1A) and define the functional human-specific duplications. All daughter duplications are approximately 10–11 kbp proximal upstream of an *NPBF* gene, which has been implicated as a partner in creating *NOTCH2NL* fusion genes (Figure 1A) (Fiddes et al. 2019). While all *NOTCH2NL* genes structurally have five exons, in T2T-CHM13 *NOTCH2NLA* and *NOTCH2NLC* gene models are distinct from the other paralogs because their first exon is suggested by gene annotation to be untranslated (Figure 1B), due to unique mutations that remove the canonical *NOTCH2* initiator methionine and secretory signal (Fiddes et al. 2018). However, it has been suggested in both paralogs that a non-standard CTG initiation allows transcription and translation of nearly the entire canonical protein (Lodewijk et al. 2020). Unlike the previous assembly, GRCh38, we find that *NOTCH2NLB* shares the same initiator methionine disruption typically attributed to *NOTCH2NLA*. A striking structural difference between T2T-CHM13 and GRCh38 is a nearly 2.5 Mbp inversion of sequence around *NOTCH2NLB*, which changes the orientation of the gene (Supplementary Figure 1).

To characterize the extent of synteny among the *NOTCH2NL* SDs, we performed a self-alignment of T2T-CHM13 defining the length and percent identity of each paralogous locus (Methods) to all others (Table 1, Figure 1C). We find the largest stretch of synteny (~557 kbp) occurs between *NOTCH2NLB* and the ancestral copy *NOTCH2*. However, the most identical stretch and second largest segment of shared sequence is between *NOTCH2NLA* and *NOTCH2NLB* (99.7% identity over 416 kbp). Across all *NOTCH2NL* paralog regions the average longest length of synteny and percent identity is ~287 kbp and 99.4%. Each *NOTCH2NL* copy has unique single-nucleotide variants that distinguish copies on T2T-CHM13 but the high degree of sequence identity over large swaths of the genome creates ample opportunities for IGC as well as non-allelic homologous recombination (NAHR) (Dumont and Eichler 2013, Vollger et al. 2022, Vollger et al. 2023, Sharp et al. 2006) in other human haplotypes.

We hypothesized that using the larger sequence context surrounding each paralog would uniquely identify the reference position of each *NOTCH2NL* gene due to the accumulated pattern of duplications/duplicons over time in each paralog’s history (Marques-Bonet and Eichler 2009). We, therefore, used the order and orientation of duplicons flanking each *NOTCH2NL* copy as defined by DupMasker (Jiang et al. 2008) to generate a “barcode” of each locus to readily identify regions in other human genomes when compared to T2T-CHM13 (Figure 1B, Methods). Representing the surrounding sequence in terms of the higher-order duplication content over 1 Mbp regions helped define orthologous locations in the presence of the homogenizing effects of IGC (Vollger et al. 2023).

Specific pairs of paralogs show extended homology (Figure 1C) as well as structural differences of potential functional consequence. *NOTCH2* and *NOTCH2NLR,* for example, show extended homology that is maintained between *NOTCH2NLA* and *NOTCH2NLB*, where synteny extends even farther into the corresponding *NBPF* region that follows the *NOTCH2NL* copies. Importantly, all copies of *NOTCH2NL* show one breakpoint with respect to the ancestral *NOTCH2* corresponding to the *NBPF* duplicon that demarcates the 3’ end of each derived-duplicated gene (Figure 1C). Additionally, the presumptive pseudogene *NOTCH2NLR* is missing the upstream nongenic ancestral *NOTCH2* sequence present in *NOTCH2NLA* and *NOTCH2NLB*. *NOTCH2NLC* appears the most structurally divergent; most paralogs share the bulk of synteny upstream of the gene model, yet the opposite is true for *NOTCH2NLC*, which has the most homologous alignments with *NOTCH2NLR*.

### Independent *NOTCH2NL* duplications and large-scale restructuring of ape chromosome 1

While partial duplications of *NOTCH2NL* had previously been noted in chimpanzee and gorilla (Fiddes et al. 2018), characterization of these ape copies was largely incomplete because the regions were not fully resolved in earlier primate genome assemblies. We took advantage of the recent release of finished NHP genomes (Mao et al. 2024, Yoo et al. 2025), to develop a more complete framework for *NOTCH2NL* evolution. SDs of the *NOTCH2* locus are observed in both haplotypes of chimpanzee, gorilla, and bonobo but not in orangutan or macaque. In total, for each primary assembly (haploid) we identified 26 distinct *NOTCH2NL* SDs among nonhuman apes (NHA) (average of 215 kbp). Though the difference in average SD lengths between NHA and human (311 kbp) are not significant (p=0.21; t-test one-sided), NHAs lack extensive blocks of synteny with the regions flanking *NOTCH2NL* paralogs in humans (Supplementary Figure 2B). Within NHA SDs we identified 26 *NOTCH2NL*-sequence-containing gene homologs in chimpanzee (n=9), bonobo (n=10), and gorilla (n=7) in addition to *NOTCH2*, which makes a total of 29 *NOTCH2/NL* sequences (Table 2). Consistent with Fiddes et al. (2018), all NHA homologs appear truncated with respect to *NOTCH2*. All are missing different canonical exons relative to the known human *NOTCH2NL* gene models (Figure 1B).

**Figure 2.**
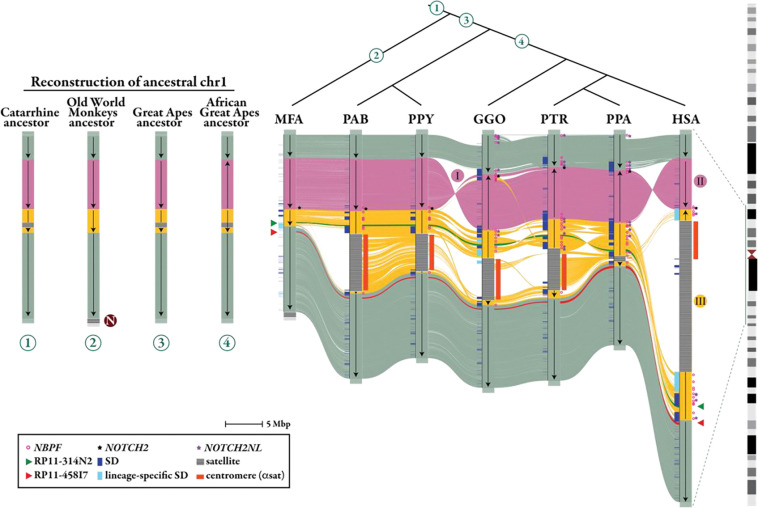
Ape evolutionary rearrangement and expansion of human chromosome 1p21.2-q23.2. The genomic structure of chromosome 1p21.2-q23.2 region is compared among macaque (MFA), Sumatran orangutan (PAB), Bornean orangutan (PPY), gorilla (GGO), chimpanzee (PTR), bonobo (PPA), and human (HSA) with annotations that include ancestral *NOTCH2* (black stars), *NOTCH2NL* duplications (purple stars), *NBPF* duplications (pink circles), and the centromere (orange bars). The circled numbers represent previous ancestral states of chromosome 1. Three distinct evolutionary inversions are predicted (I, II, III). Two probes (RP11–314N2, green, and RP11–458I7, red) used in FISH analyses from Szamalek et al. (2006) are shown (green and red triangles). Both probes map to the q-arm in humans, with the green probe located inside the inverted region and the red probe outside. FISH data from Szamalek et al. (2006) revealed that in chimpanzee the green probe maps to the region homologous to the human p-arm, while the red probe maps to the q-arm. Sequence analysis supports the FISH mapping and shows that in great apes the sequence of the two probes (represented as red and green lines in the SVbyEye) map on opposite sides of the centromere.

Next, we investigated the long-range synteny and structural changes that occurred in conjunction with the SDs in each ape lineage. The genomic organization of the chromosome 1p21.2-q23.2 region was compared within the context of a generally accepted primate phylogeny, including macaque, Sumatran and Bornean orangutans, gorilla, chimpanzee, bonobo, and human (Figure 2). Sequence comparison together with data from previous findings (Stanyon et al. 2008) suggest that the chromosomal configuration observed in orangutans represents the ancestral catarrhine state. The analysis indicates that three distinct inversion events occurred during the evolution of the chromosome 1p21.2-q23.2 region. The first inversion (I) occurred in the ancestor of African great apes, flipping the region into its current orientation in gorilla, chimpanzee, and bonobo. Subsequently, in the human lineage, this region reverted to its ancestral configuration (inversion II) but expanded in size. We estimate that this region is 17 Mbp larger than the syntenic region in chimpanzee and orangutan. Additionally, an expansion of SDs in humans coincided with a human-specific pericentric inversion (III), corresponding to the rearrangement originally described by Yunis and Prakash (Yunis and Prakash 1982) and subsequently refined to 154 kbp and 562 kbp breakpoint intervals at chromosome 1p11.2 and 1q21.3, respectively (Szamalek et al. 2016). In humans this event uniquely positioned *NOTCH2NLA*, *NOTCH2NLB*, and *NOTCH2NLC* on the long arm of chromosome 1, splitting the *NOTCH2NL* locus across the centromere when it had previously always existed on a single chromosome arm. The region encompassing *NOTCH2NL* paralogs has undergone significant restructuring via duplication and inversion among all great apes, but especially in the human genome.

In addition to the inversions, structural variants, including 308–829 insertions and 312–816 deletions (>50 bp), were observed across the NHAs (Supplementary Table 2), significantly enriched at the breakpoints of inversions (*p*<0.05), in chimpanzee, bonobo, and gorilla (Supplementary Table 2). Assessing the region in terms of duplicated sequences, we also found that the locus is enriched with SDs (*p*<0.001, in T2T-CHM13 space); the largest number of duplicated sequences was identified in gorilla (7.1 Mbp) followed by *Pan* (chimpanzee and bonobo species) lineage (6.5–6.6 Mbp) and orangutans (2.6 Mbp). Quantifying the overall structural events associated with genic regions, we identified 9–15 of insertions, deletions, and SDs overlapping with *NOTCH2/NOTCH2NL*.

**Figure 3.**
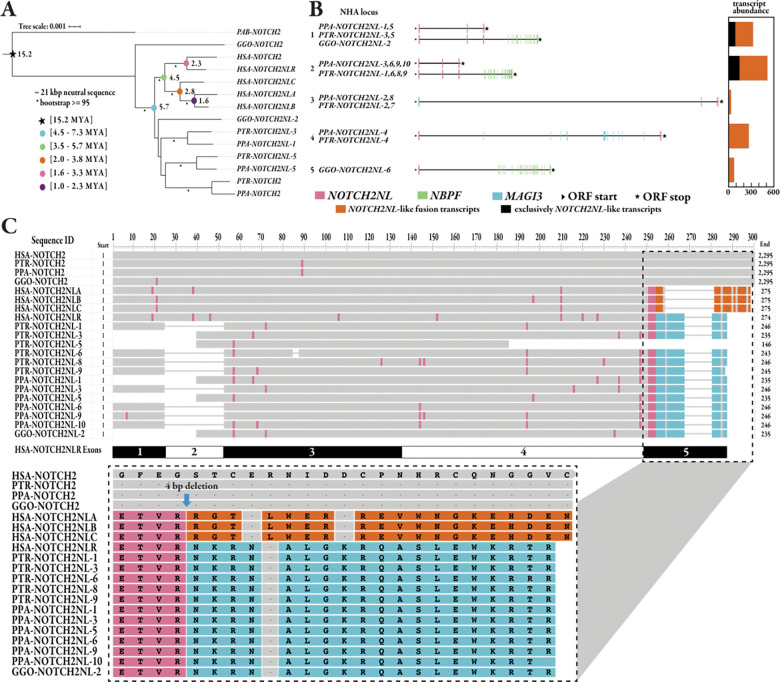
*NOTCH2/NOTCH2NL* phylogeny. **a)** A maximum likelihood phylogeny based on a multiple sequence alignment of 21 kbp of intronic *NOTCH2/NL* sequence from a subset of paralogs of five ape species, using Sumatran orangutan as an outgroup. Bootstrap support (>95%) is indicated (asterisk). Estimated divergence times of human paralogs and their confidence intervals are indicated (multicolored dots). Timings were based on human–orangutan divergence time of 15.2 MYA (Methods). **b)** Examples and abundance of five transcript types, which are representative of 20/26 *NOTCH2NL*-like loci in NHA from testis, fibroblast/lymphoblastoid cell lines, iPSCs, neuroepithelium, and neural progenitor cells. The histogram to the right of each model represents the number of Iso-Seq transcripts in support of the different predicted models at the locus. **c)** Multiple sequence alignment (MSA) of predicted protein sequences from 13/26 NHA NOTCH2NL-like loci, NOTCH2 from the NHAs, and all five NOTCH2/NL paralogs from human. Pop-out of exon 5 alignment shows NHAs possess the same unmodified carboxy terminus as NOTCH2NLR, which lacks a 4 bp deletion necessary for expression (Fiddes et al. 2018).

Next, we constructed a maximum likelihood (ML) phylogeny using shared intronic sequence (intron 2) from a subset (8/29) of African ape *NOTCH2/NL* paralogs, the five human paralogs, and *NOTCH2* from Sumatran orangutan (Methods). We observe a distinct monophyletic clade populated only by the human T2T-CHM13-*NOTCH2NL* paralogs (Figure 3A), suggesting independent duplication or recent human-specific IGC. The topology of the tree further suggests independent expansions in the gorilla and *Pan* ape lineages. In contrast, bonobo and chimpanzee share ancestral copies prior to their divergence (1–2 MYA) (Yoo et al. 2025). All species harbor four shared *NOTCH2NL* homologs, while three additional homologs are specific to gorilla, two are specific to the *Pan* genus, and three are specific to chimpanzee and bonobo, respectively. We also constructed a tree on the smaller intron 3 that includes 28/29 NHA *NOTCH2/NL* sequences, five human paralogs, and *NOTCH2* from Sumatran orangutan, albeit with lower bootstrap support. This phylogeny also supports recurrent expansions of *NOTCH2NL* in humans, the *Pan* genus, and gorillas (Supplementary Figure 3).

Using orangutan as an outgroup, we estimate the timing of the duplications or IGC events (Methods) in the human lineage (Figure 3A). Our analysis predicts that the human-specific *NOTCH2NL* copies emerged early in human evolution, around 4.5 MYA (3.5–5.7 MYA), soon after African ape speciation, and that such duplications were also occurring among the other ape lineages (albeit independently or subsequently derived from a larger initiating ancestral ape duplication). Approximately 2.8 MYA (2.0–3.8 MYA), the human-specific copies begin to diverge, distinguishing *NOTCH2NLC* from *NOTCH2NLA/B. NOTCH2NLA* and *NOTCH2NLB* appear to have diverged around 1.6 MYA (1.0–2.3 MYA), although once again IGC may have homogenized these loci since there is ample evidence of ongoing gene conversion (see Patterns of NOTCH2NL human genetic variation) in present day humans for these two copies, which map in closest proximity to one another.

### NHA *NOTCH2NL* copies and fusion transcripts

The completion of NHA genomes (Yoo et al. 2025) also allowed for a more detailed investigation of *NOTCH2NL*-like homologs in chimpanzee, bonobo, and gorilla when compared to previous analyses using much less complete genomes (Fiddes et al. 2018). Like in humans, nearly all homologs in NHA map 11–12 kbp upstream of *NBPF* genes (25/26) (Supplementary Table 3). We performed an additional duplicon analysis of the *NBPF* genes downstream of *NOTCH2NL* paralogs/homologs in human and NHA (Supplementary Figure 5). We confirmed that the human *NBPF* copies downstream of *NOTCH2NLA/B/C* have a large expansion of three Olduvai domain copies: HLS1, HLS2, and HLS3. These domains appear in ordered groups of three with 10–14 triplets occurring in these *NBPF* copies. (Fiddes et al. 2019, Pacheco et al. 2023). These triplets are found in low numbers in some NHA *NBPF* genes (1–2 triplets) as well as the *NBPF* copy downstream of human *NOTCH2NLR*, which has a high number of these three domains but only two triplets (Fiddes et al. 2019) (Supplementary Figure 5). In contrast, all the NHA *NBPF* genes have a high copy number of the CON1, CON2 and CON3 Olduvai domains.

We leveraged long-read RNA sequencing (RNA-seq) data from testis and fibroblast/lymphoblastoid cell lines from chimpanzee, bonobo, and gorilla (Yoo et al. 2025); iPSC and neuroepithelium from chimpanzee (Pollen Lab, unpublished); and neural progenitor cells from bonobo (Pollen Lab, unpublished) to search for evidence of transcriptional support for all 26 *NOTCH2NL*-like NHA homologs. In contrast to previous studies (Fiddes et al. 2018), we were able to annotate valid open reading frames (ORFs) across the majority of *NOTCH2NL*-like loci (23/26) in a set of NHA transcripts. While most transcripts predict fusions of *NOTCH2NL* with other genes (24/26), some transcripts (14/26) predict proteins most similar to human *NOTCH2NLR*-like sequence with ORF lengths ranging between 235–246 amino acids (AAs). These predicted proteins are similar (p=0.1; t-test one-sided) to human *NOTCH2NL* ORF lengths (236–274 AAs) (Supplementary Table 3). We confirmed that these SDs and gene copies are present in the alternate haplotype of each NHA individual with a small amount of expected copy number variation due to the complexity and large number of duplications in the region (n=9 in chimpanzee, n=9 in bonobo, and n=9 in gorilla). Of these, four copies in the primary gorilla assembly and six in the gorilla alternative assembly represent extremely small fragments of the *NOTCH2*/*NL* genes.

The vast majority (19/26) of these NHA *NOTCH2NL*-like loci create fusion transcripts between *NOTCH2NL* at the 5’ end and *NBPF* at the 3’ end (Figure 3B), which is similar to what is seen in all human paralogs (not including *NOTCH2*), but especially in human *NOTCH2NLR* (see Transcriptional expression and protein stability of NOTCH2NL paralogs). We see copies that are fused with other genes as previously reported (Fiddes et al. 2018), such as *MAGI3* in chimpanzee and bonobo and *BRD9* in gorilla. We also discovered unreported gene fusions between *NOTCH2NL* and other genes, like *LRIG2* and *SORT1* (Supplementary Table 3). All *NOTCH2NLR*-like transcripts in NHAs differ from the human transcripts because they have lost either exon 1 (containing the secretory pathway signal sequence) or exon 2 (Figure 3B). The phylogeny, different duplication architecture, and varying gene structures all support a largely independent evolutionary expansion among the great apes. Humans appear to be the only species with *NOTCH2NL* transcripts that are predicted to make a stable protein, likely because NHA copies lack the 4 bp deletion that was found to be essential for *NOTCH2NLA/B/C* protein expression (Fiddes et al. 2018). We confirm this 4 bp deletion, which is after the fourth AA in the fifth exon, modifies the final 19–20 AAs of the carboxy terminus in not just a paralog-specific, but also human-specific, fashion (Figure 3C). However, we cannot definitively comment on the functional role of NHA transcripts, most of which have strong Iso-Seq support (minimum of five and an average of 30 transcripts per predicted transcript model). NHA transcripts also maintain reasonable ORFs ranging between 235–246 AAs for *NOTCH2NLR*-like transcripts and 783–4694 AAs for fusion transcripts.

### Patterns of *NOTCH2NL* human genetic variation

To understand human *NOTCH2NL* genetic variation, including structural differences among human haplotypes, we initially selected 94 haploid genome assemblies recently generated by the HPRC (Liao et al. 2023). Despite long-read sequencing advances, regions encompassing *NOTCH2NL* are still challenging to assemble because the length and high degree of sequence identity of the paralogs (Table 1) leads to misassembly and collapse (Porubsky et al. 2023). We, therefore, manually evaluated the accuracy of the HPRC assemblies across the *NOTCH2NL* loci using a combination of mapping and assembly validation tools (minimap2, SafFire, and NucFreq) (Li et al. 2018, Vollger et al. 2022, Vollger et al. 2018) (Methods). A proportion of samples were reassembled using Verkko (Rautiainen et al. 2023, Guitart et al. 2024) and then validated successfully. To validate, we assessed contiguity, annotated gaps, examined for the presence of collapses, and verified true structural variants. Of these assemblies, 69 (73%) passed QC for sequence and structural accuracy; 54% of these genomes were of African origin while the remaining 46% were of non-African origin representing in total 14 distinct population groups (Supplementary Table 4).

**Figure 4.**
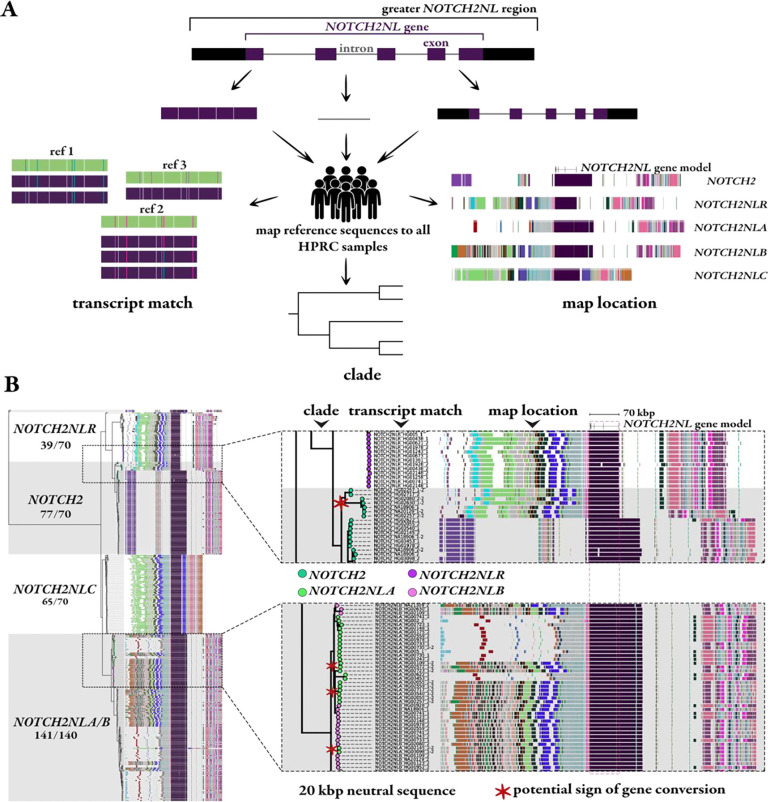
Patterns of human *NOTCH2NL* structural variation and gene conversion. **a)** Workflow to characterize *NOTCH2NL* paralog identity based on i) best transcript match (defined as the fewest mismatches with respect to T2T-CHM13 reference CDS annotation), ii) phylogenetic clade (assignment to nearest monophyletic grouping based on *NOTCH2* intronic ML tree), and iii) map location (defined here as the long-range genomic context based on DupMasker barcodes). **b)** Analysis of 70 human haplotypes depicts the clade assignment based on the phylogenetic tree, then the best transcript match, and finally the long-range duplicon organization based on the assembled HPRC genomes. Disagreements in paralog identity suggest potential gene conversion; examples marked with red asterisks.

**Figure 5.**
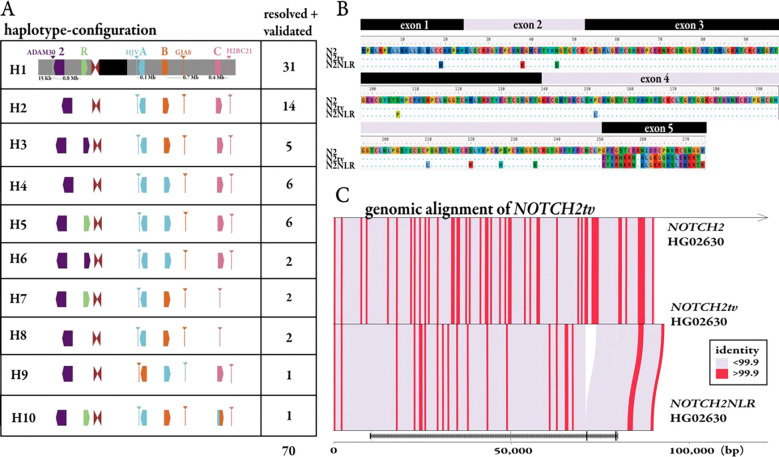
*NOTCH2NL s*tructural diversity and *NOTCH2tv.* **a)** A simplified schematic summary of the *NOTCH2NL* haplotype organization and frequency based on 66 sequence-resolved HPRC genomes and the T2T-CHM13 reference. **b)** Alignment of predicted AAs for the three paralogs suggests that *NOTCH2tv* arose as a result of an interlocus gene conversion (IGC) of *NOTCH2NLR* from *NOTCH2.* **c)** Nucleotide alignment of *NOTCH2tv* (middle) to ancestral *NOTCH2* (top) and *NOTCH2NLR* (bottom) confirms larger stretches of near perfect sequence identity (red≥99.9%) between *NOTCH2tv and NOTCH2*, consistent with IGC.

Among these 69 genomes, we distinguished 10 different structural configurations operationally defining H1 based on the T2T-CHM13 reference configuration described above (70 haplotypes in total). Given the anticipated high degree of IGC (Fiddes et al. 2018, Vollger et al. 2022), we developed a tripartite workflow (Figure 4A) to assign *NOTCH2NL* identity. First, we examined the best transcript match by identifying which *NOTCH2NL* coding sequence best matches *NOTCH2NL* copies assigned in the T2T-CHM13 reference (Methods). Second, we used *NOTCH2NL* intronic sequence to construct a tree identifying a phylogenetic framework for each *NOTCH2NL* haplotype assigning different haplotypes to related clades. Third, we used the extended duplication organization as defined by the DupMasker barcode described above (Figure 1B) to examine the long-range organization of the region flanking *NOTCH2NL*. The combination of these results (Figure 4B) was used to delineate IGC events and to further define 10 distinct human haplotype configurations (H1-H10) (Figure 5A).

Of the haplotype-resolved genomes, 43% share the canonical haplotype configuration (H1) observed in T2T-CHM13, thus, representing the major human haplotype (Figure 5A). Among the remaining nine configurations, seven are observed more than once in this subset from the HPRC (Figure 5A). Notably, the haplotype configuration currently represented in the standard human reference, GRCh38, is characterized by a nearly 2.5 Mbp inversion that reverses the orientation of *NOTCH2NLB* relative to T2T-CHM13 yet has not been observed in any other human haplotype. GRCh38, thus, either represents a minor variant or a misassembly. Our analysis indicates that the *NOTCH2NLR* pseudogene is not present in 43% (30/69) of all haploid assemblies (H2–4, H6, and H8–9). H2, H4, H8, and H9 configurations strictly represent deletion events, which occur in 30% (21/69) of haploid assemblies and are a much more common occurrence than previously estimated (8%) (Fiddes et al. 2018). The additional depletion of *NOTCH2NLR* paralogs represent a novel IGC event between *NOTCH2* and *NOTCH2NLR* (H3 and H6). Aside from the *NOTCH2*-like IGC events, the metazoan conserved developmental ancestral gene, *NOTCH2*, is invariant with respect to copy number; however, so too is *NOTCH2NLA*, which is present in all sequenced human haplotypes. *NOTCH2NLC* is deleted in four haploid assemblies with two different configurations (H7 and H8) while in another (H10) it appears to have been converted to a *NOTCH2NLA/B* hybrid. Other than pseudogene copies, *NOTCH2NLB* appears to be the most copy number polymorphic with respect to its identity, due to IGC between *NOTCH2NLA* and *NOTCH2NLB*. Yet, *NOTCH2NLA* and *NOTCH2LB* are very similar in structure and the sum of these two is constant in all human haplotypes (n=2).

To reach these configurations, we leveraged the disagreement of mapping location and transcript/coding sequence matches identified through the paralog identity workflow to more systematically assess and characterize gene conversion events of potential functional consequence that were noted above. For example, a *NOTCH2NL* paralog with the same mapping location as *NOTCH2NLB*, but a coding sequence that is identical to *NOTCH2NLA*, defines an IGC event between *NOTCH2NLA* and *NOTCH2NLB* resulting in two copies of *NOTCH2NLA*. Overall, in this study, 20% (14/69) of haplotypes (H4, H5, H6) appear to have a *NOTCH2NLB* to *NOTCH2NLA* conversion event (Figure 5A). This IGC event is confirmed by our workflow where both the phylogeny and duplication barcode are disrupted between *NOTCH2NLA* and *NOTCH2NLB* (Figure 4B). The analysis suggests that *NOTCH2NLA* is the preferred donor locus as we never observe *NOTCH2NLA* converted to a *NOTCH2NLB* identity.

As mentioned above, during this analysis we observed a second gene conversion event that had not been previously identified: direct conversion of the *NOTCH2NLR* pseudogene from the *NOTCH2* ancestral locus (Figure 4B), which is 654 kbp distant. Ten percent of haplotypes (H3 and H6) harbor a copy of the gene at this locus that resembles a truncated version of *NOTCH2* rather than *NOTCH2NLR*; this includes two H6 haplotypes that exhibit both gene conversion events (making the combined amount of IGC across haplotypes 28% instead of 30%). As a result, all eight AA changes associated with NOTCH2NLR now match the ancestral NOTCH2 (Figure 5B). When surveying gene conversion at the gene level, we see more >99% identity bins between *NOTCH2* and the gene conversion product than between the product and *NOTCH2NLR* (Figure 5C). Notably, the H3 and H6 haplotypes are significantly enriched in African samples (p=0.007, Fisher’s exact test), suggesting *NOTCH2NLR* to *NOTCH2* could be an ancestral gene conversion event. Because of its sequence similarity to *NOTCH2*, we renamed this version of *NOTCH2NLR* to *NOTCH2tv* (*NOTCH2*-truncated-version), a novel sixth paralog in the gene family.

### Accessible chromatin architecture surrounding the *NOTCH2NL* paralogs

Having established the genetic architecture of *NOTCH2NL* paralogs and their surrounding loci, we next sought to determine how the structure of these SDs influences the gene regulatory landscape surrounding *NOTCH2NL* paralogs. Gene regulatory landscapes are often defined using techniques like ATAC-seq and DNase-seq (Buenrostro et al. 2013, Thurman et al. 2012), which can detect accessible chromatin elements. However, SDs have been historically excluded from these short-read-based techniques, as the sequence reads are largely impossible to unambiguously assign to large, highly identical SD regions. Fiber-seq, in contrast, is a long-read-based approach for mapping chromatin architecture (Stergachis et al. 2020) and we previously demonstrated that this approach can be used to map chromatin accessibility to complex genomic regions, such as SDs (Vollger et al. 2024).

To determine whether there are any accessible chromatin elements within the vicinity of *NOTCH2NL* paralogs, we mapped CHM13 Fiber-seq data (Dubocanin et al. 2022) to the 300 kbp regions surrounding each *NOTCH2NL* paralog transcription start site (TSS) in T2T-CHM13 and compared these accessible chromatin maps from each paralog based on genetic synteny (Supplementary Figure 6). Overall, this revealed that each *NOTCH2NL* paralog shares a promoter with accessible chromatin within CHM13 cells, and that each has between 503 and 634 accessible chromatin elements within 300 kbp of their TSS. Notably, each paralog shows a largely unique surrounding accessible chromatin landscape along regions that lack synteny, suggesting that non-syntenic sequence contributes to regulatory differences among the five *NOTCH2NL* paralogs.

**Figure 6.**
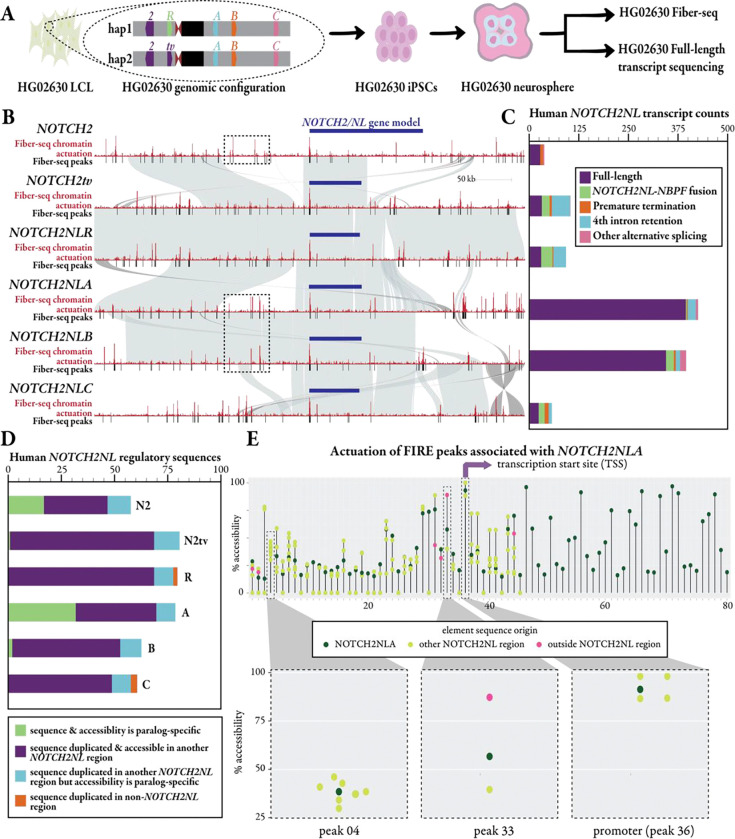
Regulatory architecture and transcription of *NOTCH2NL* in neurospheres. **a)** HG02360 was reprogrammed into iPSCs, differentiated into neurospheres, and then subjected to Fiber-seq and Iso-Seq to define putative regulatory elements and generate full-length transcripts. **b)** Fiber-seq peaks and chromatin actuation sites for each *NOTCH2/NL* paralog in the context of homology (gray), gene model, and transcription start site (TSS). Dotted black boxes are around elements specific to a region only shared across *NOTCH2*, *NOTCH2NLA*, and *NOTCH2NLB*; though the underlying sequence is nearly identical, we show paralog-specific actuation signals. **c)** The absolute abundance of full-length transcripts compared to other premature termination, fusion, and intron retention products in neurospheres. **d)** Boxplot showing categorization of accessible elements surrounding each *NOTCH2NL* paralog based on the presence of duplicate sequence and accessibility at that sequence on the different paralogs. Note that *NOTCH2* and *NOTCH2NLA* have the greatest proportion of paralog-specific sites (dark green). **e)** Percent actuation of each accessible regulatory element surrounding the *NOTCH2NLA* paralog (dark green) as well as the percent actuation of duplicate sequences for each element that are present surrounding other *NOTCH2NL* paralogs (light green) or outside of the *NOTCH2NL* paralogs (pink).

To further investigate the accessible chromatin landscape across all the *NOTCH2NL* paralogs, including *NOTCH2tv*, in more relevant tissue we generated Fiber-seq and long-read full-length transcript sequencing data from neurospheres of an HPRC sample that contains *NOTCH2tv* in addition to the other four copies of *NOTCH2NL*. Specifically, lymphoblast cell lines from HG02630 were reprogrammed into iPSCs, which were then differentiated into cortical neurospheres (Figure 6A, Methods). HG02630 has both a *NOTCH2tv* haplotype and a canonical *NOTCH2NLR* haplotype, enabling the evaluation of the gene regulatory landscape of all the *NOTCH2NL* paralogs within the same individual. Overall, like CHM13, we observe that *NOTCH2*, as well as all the *NOTCH2NL* paralogs, including *NOTCH2tv*, have accessible promoter elements that show a similar degree of chromatin accessibility (Figure 6B). In contrast to the promoter, accessible elements within the vicinity of these genes, once again, exhibit marked paralog-specific patterns both in terms of their location and accessibility.

Comparison of these accessible Fiber-seq-based chromatin maps (Methods) with underlying synteny maps reveals that nearly 87% of all accessible chromatin elements within 300 kbp of the *NOTCH2NL* TSSs share duplicated sequence with at least one other *NOTCH2NL* paralog. This suggests prevalent reuse of genomic sequence with putative gene regulatory potential across these SDs (Figure 6D). Furthermore, 84% of those elements near multiple *NOTCH2NL* paralogs also show some level of accessible chromatin at more than one of their paralogous sites, demonstrating that by and large, these elements retain their ability to form accessible chromatin when rearranged in different genomic positions. Overall, the accessible chromatin landscape surrounding each *NOTCH2NL* paralog appears predominantly populated by these multi-paralog accessible chromatin elements. However, these paralogs often diverged in their magnitude of chromatin accessibility (Figure 6B, E) as well as their rearrangement relative to the *NOTCH2NL* promoters, indicating that position effects within these different SDs may impact these putative regulatory elements in a quantitative manner as opposed to simply abrogating their chromatin accessibility.

We also observe that 10% of all accessible chromatin elements within 300 kbp of the *NOTCH2NL* TSSs map to duplicated sequence on a different chromosome. Five elements exclusively share duplicated sequence with regions on a different chromosome (about 1% of all elements) (Figure 6D). This suggests that the creation of the *NOTCH2NL* SDs was associated with the potential repurposing of accessible chromatin elements from elsewhere in the genome.

In total, we find that 12% of all accessible chromatin elements surrounding *NOTCH2NL* paralogs are specific to only one paralog. These paralog-specific unique elements are concentrated in the *NOTCH2* and *NOTCH2NLA* regions. Notably, these paralogs are the most fixed for copy number variation within the human population.

### Transcriptional expression and protein stability of *NOTCH2NL* paralogs

We found distinct differences within the transcript abundance of each of the *NOTCH2* paralogs (Figure 6C), indicating that these paralog-specific accessible chromatin elements may be creating unique gene regulatory environments for each of the *NOTCH2* paralogs. Specifically, *NOTCH2NLA* and *NOTCH2NLB* had ~3-fold higher steady-state transcript abundance than the other *NOTCH2* paralogs. Furthermore, we observed that although the promoter and transcript sequence of *NOTCH2tv* mirrors that of *NOTCH2*, the transcript abundance and composition of *NOTCH2tv* appeared to mirror most closely that of *NOTCH2NLR*. Specifically, *NOTCH2tv* and *NOTCH2NLR* had 102 and 91 transcripts, respectively. However, only ~30% of these transcripts represented canonical full-length transcripts, with the majority arising from fusion transcripts, and an incorrectly spliced d exon. Surprisingly, the fusion transcripts of all *NOTCH2NL* copies do maintain ORFs predicted to be 1179–1574 AA long. Overall, this indicates that despite the transcript identity of *NOTCH2tv* matching the first four exons of *NOTCH2*, the surrounding gene regulatory architecture in fact mirrors that of *NOTCH2NLR*, potentially impacting the overall function of *NOTCH2tv.* This is likely a result of the gene conversion event being bounded by a 75 kbp syntenic block between *NOTCH2* and *NOTCH2NLR* that spans from just upstream of their promoters to their fourth introns.

Although the gene conversion event results in NOTCH2tv adopting the exact same protein sequence as NOTCH2 for its first 250 AAs, NOTCH2tv ends in a distinct 23 AA sequence arising from its terminal fifth exon. NOTCH2NLR was previously shown to form an unstable protein product, which is thought to be driven by its carboxy-terminal sequence. As such, we sought to evaluate whether NOTCH2tv similarly forms an unstable protein product, as its C-terminal sequence shares 91% AA similarity to th NOTCH2NLR. We transfected HEK293 cells with a constitutive reporter system containing either *NOTCH2tv*, *NOTCH2NLR*, *NOTCH2NLB*, or a negative control and demonstrated that despite the ability of *NOTCH2tv*, *NOTCH2NLR*, and *NOTCH2NLB* to produce sufficient transcripts in this reporter, only *NOTCH2NLB* resulted in a stable protein product (Supplementary Figure 7). Together, these data indicate that although gene conversion has generated a new paralog of *NOTCH2* that contains sequence and promoter features consistent with *NOTCH2*, this new paralog retains the overall gene regulatory architecture and transcript patterns of *NOTCH2NLR* and is similarly unable to form a stable protein product and, thus, likely represents a pseudogene.

## DISCUSSION

The rapid expansion of interspersed SDs in the ancestral genome of African apes around 8–15 MYA (Marques-Bonet et al. 2009) provided the substrate for the human genome to evolve both ape and species-specific genes. *NOTCH2NL* is one of at least five human- and ape-specific SD gene families that have been implicated in expansion of the human frontal cortex. This includes genes associated with delayed maturation of synapses and increasing synaptic density (*SRGAP2C*) (Charrier et al. 2012, Schmidt et al. 2019), genes like *NOTCH2NL* (Fiddes et al. 2018, Suzuki et al. 2018) implicated in cortical progenitor self-renewal, and genes directly promoting cortical and basal progenitor amplification (*TBC1D3*, *ARHGAP11B*, and *CROCCP2*) (Ju et al. 2016, Florio et al. 2015, Fischer et al. 2022, Van Heurck et al. 2023). Like *NOTCH2NL* it is noteworthy that most of these human-specific gene innovations originated from an incomplete duplication that truncated the ancestral gene model, leading to novel human-specific isoforms. In fact, the incomplete duplication appears to have been a first critical step in either neofunctionalization (*ARHGAP11B*) or dominant negative effects (*SRGAP2C*, *NOTCH2NL*) where shorter, derived proteins either interfere or modulate ancestral protein function through protein-protein interactions (Florio et al. 2015, Fischer et al. 2022, Charrier et al. 2012, Dennis et al. 2017, Fiddes et al. 2018, Suzuki et al. 2018, Schmidt et al. 2019).

We demonstrate that *NOTCH2NL*, like other recently characterized primate gene families (*TBC1D3*, *LRRC37*, and *NPIP*) (Guitart et al. 2024, Giannuzzi et al. 2013, Cantsilieris et al. 2020, Dishuck et al. 2025), likely independently expanded in human, chimpanzee, and gorilla. The independent expansion of *NOTCH2NL* among the apes was first suggested by Fiddes et al. (2018), based on sequencing data showing African apes had gene truncations of differing lengths. Our phylogenetic and comparative synteny analysis of T2T ape assemblies generally confirms recurrent duplications although we cannot preclude an originating larger duplication in the ape last common ancestor (e.g., *NOTCH2NLR*) that was subsequently restructured differentially through IGC and rearrangement in the different ape lineages (Figure 3A). The basis for this recurrence or genomic instability is unknown but it is interesting that most *NOTCH2NL* ape copies are also associated with the *NBPF* duplicon—an association that is postulated to have co-evolved both in terms of structure and transcriptional regulation (Fiddes et al. 2019). *NBPF* is one of about a dozen core duplicons (along with *TBC1D3*, *LRRC37*, and *NPIP*) implicated as a potential driver of interspersed SDs in the primate lineage (Jiang et al. 2007, Dumas and Sikela 2009, O’Bleness et al. 2012). Of note, a comparative analysis of *NBPF* associated with *NOTCH2NL* reveals species-specific expansions of different portions of the *NBPF* Olduvai in different apes (Supplementary Figure 5), so it is possible that *NBPF* plays a more general role in gene innovation in ape species other than human.

Notwithstanding this proclivity to duplicate in the common ancestor of great apes, the apparently functional human copies of *NOTCH2NL* arose much later in human evolution. We estimate that the human-specific expansions (or IGC events) occurred around 4.5 MYA and diversified over a range of 2.8–1.6 MYA (Figure 3A). It is interesting to note that other duplicate gene families implicated in the expansion of the human frontal cortex (*SRGAP2C* and *TBC1D3*) show similar evolutionary trajectories beginning to emerge 2–3 MYA (Dennis et al. 2012, Guitart et al. 2024). This is significant in the context of fossil record evidence, which suggests divergence of the genus *Homo* from *Australopithecus* ~2 MYA and a subsequent initial increase in archaic hominin cranial volume. There is also evidence of subsequent increases in cranial volume taking place between 2.0–1.5 MYA consistent with the diversification of *NOTCH2NL* genes in humans (Tattersall et al. 2023).

Using a large collection of contiguously assembled reference genomes, we observed eight different strongly supported *NOTCH2NL* haplotype configurations and a high amount of population diversity compared to lower identity and fixed copy gene families (Figure 5A), which is often the case for SDs (Dennis et al. 2017, Vollger et al. 2022, Vollger et al. 2023). We also encounter polarized signals of gene conversion, suggestive of selection. This seems especially significant in the case of *NOTCH2NLA*, which is the only paralog present in all assemblies and has even expanded, seemingly at the expense of *NOTCH2NLB*. In Fiddes et al. (2018), it was also postulated that having a combined dosage of A/B was more important than having two of each paralog. Like *SRGAP2C* (Dennis et al. 2012), *NOTCH2NLA r*epresents the most fixed paralog suggesting functional constraint. Gene birth can be accomplished through duplication (Ohno 1968), but there is a gap in the research on how IGC may influence this process. When first investigating the novel paralog *NOTCH2tv*, we hypothesized it was a case of gene conversion reviving a nonfunctional gene since so far *NOTCH2tv* has acquired a promoter and 4-exon N-terminus identical to *NOTCH2*. This in theory could enable *NOTCH2tv* to regulate expression similar to *NOTCH2* (Dougherty et al. 2018). However, whereas *NOTCH2NLA/B/C* contain a protein-stabilizing 4 bp deletion in their terminal exon (Figure 3C), neither *NOTCH2NLR* nor *NOTCH2tv* has this same 4 bp deletion in its terminal exon (Figure 5B). Consistent with this, we found that like *NOTCH2NLR*, *NOTCH2tv* does not produce a stable protein (Supplementary Figure 7) and, thus, is not a fully functional paralog.

One of the major known features driving SD paralogs to adopt distinct gene regulatory architectures is via large-scale alterations in the syntenic DNA content surrounding different SD paralogs. For example, the human-specific gene *HYDIN2* acquired 5’ duplicated segments that drive expression in the brain, which is a tissue that the ancestral *HYDIN* is not expressed in (Dougherty et al. 2017). Likewise, the divergent expression profiles of *CD8B* and *CD8B2* have been attributed to *CD8B2* retaining syntenic sequences that encompass only two of the elements within a six-enhancer cluster that drive *CD8B* expression (Kioussis and Ellmeier 2002). By leveraging Fiber-seq, we identify marked paralog-specific gene regulatory patterns surrounding each *NOTCH2NL* paralog. Overall, we find that the different *NOTCH2NL* paralogs frequently retain duplicated sequences that encompass an accessible chromatin element on at least one paralog. However, ~14% of elements present within these duplicated sequences exclusively show chromatin accessibility in only one paralog. Furthermore, even for those elements that do show some chromatin accessibility across two or more duplicate sequences, we find that the degree of chromatin accessibility can vary quite substantially between the two duplicates (Figure 6E). This suggests that putative regulatory elements within SDs are being subjected to positional effects, with the predominant effect being quantitative differences in chromatin accessibility as opposed to drastic changes to on/off actuation.

In summary, we hypothesize that the dramatic restructuring of the *NOTCH2NL* loci during human evolution led to the only ape lineage with functional copies. This was made possible by a dynamic set of large- and small-scale changes associated with NAHR, recurrent duplication/deletions (Sasaki et al. 2010), and IGC (Vollger et al. 2023). Many genes embedded in these regions, including *NOTCH2NL*, are associated with neurologic and developmental phenotypes, including copy number variation syndromes, such as 1q21.1 distal duplication/deletion syndrome (Mefford et al. 2008) or TAR syndrome (Klopocki et al. 2007). The fact that this region is among the most frequently rearranged regions of the human genome (Cooper et al. 2007) is a testament to the evolutionary instability that continues to persist in the human population and these changes have phenotypic consequences. Consistent with the core duplicon hypothesis that was proposed (Marques-Bonet and Eichler, 2009), the mutational lability of chromosome 1q21.1 and the emergence of novel *NOTCH2NL* genes represents a significant trade-off of selective forces during human evolution. In the case of *NOTCH2NL*, we hypothesize that the benefits of expanding the cortex must have outweighed the mutational burden of increasing the proportion of high-identity duplicated sequences in the genome. Our findings suggest that this trade-off is still ongoing. The biased gene conversion that appears to be driving the fixation of *NOTCH2NLA* and the high level of fourth intron retention among transcripts of *NOTCH2tv* and *NOTCH2NLR* may be examples of both refinement and continued evolution of a novel carboxy terminus.

## METHODS

### *NOTCH2NL*-CHM13 region identity matrix

*NOTCH2NL* paralog sequences from T2T-CHM13 V2.0 (Perez et al. 2025, http://genome.ucsc.edu) plus 1 Mbp surrounding each gene were all aligned to each other simultaneously and allowing for secondary alignments (paralog pairs with less than 1 Mbp between them had overlapping sequence removed), using minimap2 (Li et al. 2018) and the parameters

-xasm20-s1000--eqx-a--secondary=yes-p0.05

The longest nonoverlapping stretch of syntenic sequence that can be aligned between each paralog pair is represented in the matrix. The percent identity shared between each paralog pair is calculated by the number of base matches.

### *NOTCH2NL*-CHM13 region duplication barcodes

*NOTCH2NL* paralog sequences plus 1 Mbp surrounding each gene were used as input for DupMasker (Jiang et al. 2008). The .duplicons output file was processed into a .txt file to create visual tracks of duplicon barcodes for each region.

### Visual alignment of *NOTCH2NL*-CHM13 regions

*NOTCH2NL* paralog sequences plus 1 Mbp surrounding each gene were aligned to each other one by one and allowed for secondary alignments, using minimap2 parameters

-xasm20-s1000--eqx-c--secondary=yes

The output of each gene pair alignment was compiled into a single output .paf file that was used as input for SVbyEye (Porubsky et al. 2024). The ladder plot alignment figure was visualized using the plotAVA function. Each *NOTCH2NL* region’s corresponding duplication barcodes are included as an additional annotation track. The output .paf CIGAR string was also used to return a table of insertion/deletion structural variants for each paralog.

### Chromosome 1 visual alignment of *NOTCH2NL* region between human and NHPs

NHP assemblies used in this study from previous research can be found by the following GenBank accession IDs: GCA_028858775.2 (chimpanzee), GCA_029289425.2 (bonobo), GCA_029281585.2 (gorilla), GCA_028885625.2 and GCA_028885655.2 (Bornean and Sumatran orangutans) (Yoo et al. 2025), and GCA_030222085.1 (macaque) (Mao et al. 2024). Homologous sequence of the *NOTCH2NL* region in human (chr1:110,000,000–160,00,000) was identified by aligning human sequence against each of the NHP genomes. The alignment was performed using minimap2 with the parameters

-xasm20-c--eqx--secondary=no

Alignment blocks equal to or larger than 10 kbp were retained. After locating the corresponding sequences of NHPs, the alignment was performed allowing for secondary alignment using the parameter

-xasm20-c--eqx--secondary=yes

progressively in the order from human to macaque. The alignment was visualized using SVbyEye.

### NHA SD and *NOTCH2NL* analysis

SD tracks generated by Yoo et al. 2025 were used. Briefly, the SD track was annotated using SEDEF (v1.1) (Numanagic et al. 2018), after masking the repeats using TRF (v4.1.0) (Benson 1999), RepeatMasker (v4.1.5) (Tempel 2012), and WindowMasker (v2.2.22) (Morgulis et al. 2006). The SDs were filtered for length >1 kbp, pairwise sequence identity >90%, and satellite content <70%.

### *NOTCH2NL* NHA phylogeny and duplication timing

Multiple intronic sequences of *NOTCH2* were mapped to each NHA primate assembly using minimap2. The coordinates of all the *NOTCH2NL*-like regions found were used to pull out the sequence and construct a multiple sequence alignment (MSA) using MAFFT (Katoh and Standley 2013) with the parameters

--anysymbol--reorder--maxiterate1000--thread16

The MSA was then processed with IQ-TREE2, an ML phylogeny building program, and bootstrapped using the ultrafast bootstrap (Hoang et al. 2018, Minh et al. 2020). IQTREE estimated phylogenetic dating using LSD2 to build a time tree based on orangutan–human divergence time being 15.2 MYA. IQ-TREE2 was run with the parameters

--date{divergencetime_file}--keep-ident--date-tip0--date-ci100-B1000-T36

The most robustly bootstrapped tree was used in the main text.

### Annotation of *NOTCH2NL* gene fusions in NHA

NCBI RefSeq and CAT2.0. annotations from the T2T NHA genomes were used to study copies of *NOTCH2NL*-like genes across the NHA assemblies. First, the genomic annotations for any gene from the *NOTCH2NL* family were extracted from both these annotation sets. Next, any copies that were missed by either of the two methods were extracted with BLAT, using the genomic sequence of the first four exons of *NOTCH2* on the T2T NHA genomes. The full set of copies were then manually investigated on the UCSC Genome Browser with the added NHA Iso-Seq transcript data from testis, fibroblast/lymphoblastoid cell lines (available under NCBI BioProject IDs: PRJNA1016395 and PRJNA902025), as well as iPSCs, neuroepithelium, and neural progenitor cells (Pollen Lab, unpublished). The exact boundaries of the genes and fusions were determined. Blat was used to describe the exon structures of these fused genes. The predicted proteins for these copies were aligned using Clustal Omega (Sievers and Higgins, 2018) and visualized using the NCBI Multiple Sequence Alignment Viewer (1.25.3).

### *NBPF* Olduvai domain sequence analysis

The NBPF protein sequence for all the gene copies downstream of *NOTCH2NL* in human and NHA were extracted and InterPro (Blum et al. 2025) was run to annotate all protein domains on the sequences. Olduvai domains were then extracted and matched against the canonical sequences (UniProt) for HLS1, HLS2, HLS3, CON1, CON2, and CON3 using BLAT.

### Assembly validation of *NOTCH2NL* regions in HPRC assemblies

Except for T2T-CHM13, the human assemblies used in this study were originally released as a part of HPRC year 1 (Liao et al. 2023) without complete validation of every region. These assemblies are available under the Umbrella BioProject ID: PRJNA730822. To validate our set of assemblies, we first confirmed the correct assembly of contiguous sequence between *NOTCH2NL* paralogs on the same chromosome arms. This was done by evaluating the number of copies in each assembly and how many contigs they covered using rustybam (mrvollger.github.io/rustybam/). Assemblies with copies across three or more contigs were removed and those with no more than two contigs (allowing for gaps across the centromere) were assessed with NucFreq (Vollger et al. 2022). Assemblies with large collapses (excess of secondary bases in a single haplotype assembly) and large gaps (low-quality, N-based sequence) over the 1 Mbp region surrounding each *NOTCH2NL* paralog were considered incorrectly assembled. Finally, assemblies were also removed from the completed set if they were missing specific unique gene marks outside *NOTCH2NL* SD regions. Deletions were further validated using fastCN (Pendelton et al. 2018) read depth. A subset of incorrectly assembled samples was attempted to be rescued using Verkko (1.1 and 1.2) (Rautiainen et al. 2023) and added to the validated set: 69/94 haploid assemblies in total were correctly assembled and validated.

### Determining *NOTCH2NL* identity in HPRC assemblies

To determine *NOTCH2NL* identity we took a three pronged approach: (1) identifying which T2T-CHM13 *NOTCH2NL* CDS reference or ‘transcript’ best matches the *NOTCH2NL* sequence being queried using BLAT; (2) identifying the phylogenetic clade the *NOTCH2NL* sequence being queried best groups with—sequences from intron 2 of *NOTCH2/NL* from all samples queried were used to construct an MSA using MAFFT, from which an ML phylogeny was built using IQ-TREE2; and (3) identifying the mapping location of the *NOTCH2NL* sequence being queried using the greater *NOTCH2NL* region DupMasker barcode—*NOTCH2NL* intron 2 sequences plus 1 Mbp surrounding each intron were used as input for DupMasker and duplicon barcodes for each sequence were added to the tree.

### iPSC generation of HG02630

iPSCs were generated from HG02630 cultured fibroblast cells obtained from Coriell using the method described in Vollger et al. (2025).

### Making cortical neurospheres of HG02630

HG02630 iPSC line maintenance and cerebral organoid generation until day 21 were done using the methods described in Seiler et al. (2022). To achieve uniform basal ECM coating on days 6 and 7, a combination of 0.2% alginate and 0.6mg/ml Geltrex LDEV-Free Reduced Growth Factor Basement Membrane Matrix (ThermoFisher) coating was used (Hoffman et al., in preparation), and the alginate was crosslinked with CaCl2.

On day 21, the organoids were broken apart using Trypsin-EDTA (0.25%), placed in the centrifuge at 250g for 5 minutes, resuspended in 0.5 ml of PBS, and centrifuged for 5 more minutes at 250g. The supernatant was aspirated, and the cells were resuspended in 180ul of Buffer A (components missing). The sample was transferred to a PCR tube, and 180ul of 2X lysis buffer was added. Cells were spun at 350g for 5 minutes, after which the supernatant was removed. The remaining nuclei pellets were resuspended in (Buffer A, 32mM SAM, Hia4 (200U/ul)) at 25°C for 10 min. Finally, 9ul of 1% SDS was added to the sample and transferred to 1.5ml tubes using wide-bore pipette tips.

### Fiber-seq and identification of chromatin accessibility in HG02630 cortical neurospheres

PacBio HiFi Fiber-seq sequencing data were generated from HG02630 neurosphere nuclei pellets using the method described in Vollger et al. 2025. The data were analyzed using the fiberseq-FIRE pipeline (Vollger et al. 2024, Vollger et al. 2025, https://github.com/fiberseq/fiberseq-fire), which identifies single-molecule sites of chromatin actuation as well as peaks of chromatin actuation with a false discovery rate 5% threshold. Percent actuation was calculated as the percentage of fibers mapping to a given location that were classified as having a Fiber-seq Inferred Regulatory Element (FIRE).

### Long-read RNA-seq in HG02630 cortical neurospheres

PacBio MAS-seq (Al’Khafaji et al. 2024; PN: 103–072-000) data were generated from HG02630 neurosphere nuclei pellets using the method described in the RNA preparation section of the Methods in Vollger et al. (2025). The data were processed and mapped to the HG02630 diploid assembly using pbmm2 (https://github.com/PacificBiosciences/pbmm2) and isoforms were defined using the Iso-Seq pipeline (https://isoseq.how/) and annotated using Pigeon.

### Protein expression of NOTCH2tv in HEK293 cells

gBlocks were designed to contain an HA tag, *NOTCH2NL* CDS, IRES sequence, and *E-GFP* CDS and ordered using IDT (https://www.idtdna.com/page). Gibson Assembly (NEB, E5510) was used to clone constructs into a pEF-GFP vector. Each vector construct was cloned using NEB High Efficiency Transformation Protocol (C2987H/C2987I) with NEB 5-alpha Competent E. coli. Colonies were picked, inoculated, and plasmid DNA was extracted using the Monarch Plasmid Miniprep kit (NEB, T10104).

Four wells of a 6-well plate were seeded with 6.25×10^^5^ HEK293 cells and grown in DMEM (Gibco) supplemented with 10% FBS and 1% Pen-Strep at 37°C in a humidified incubator with 5% CO_2_. 24 hours after seeding, cells were transiently transfected with 2.5ug of the *NOTCH2tv*, *NOTCH2NLR*, *NOTCH2NLB*, or pEF-GFP (for GFP Ab control) expression plasmid construct using a 3:1 μl/μg ratio of Lipofectamine LTX Reagent (ThermoFisher) according to the manufacturer’s protocol. Cells were harvested 48 hours after transfection, washed in 1mL of cold PBS, resuspended in 250 ul of cold RIPA buffer (5M NaCl, 1M Tris-HCl pH 8.0, 1% NP40, 10% sodium deoxycholate, 10% SDS, 1mM PMSF, 1x Protease Inhibitor tablet (Pierce)) and incubated in a thermomixer at 4°C and 500 rpm for 20 minutes. The lysis was then spun down at 16,000 rpm for 20 minutes and the supernatant collected. 12μL of the cleared lysate was supplemented with 4μL of 4x LDS Sample Buffer (Invitrogen) and boiled at 70°C for 10 minutes. A 4–12% Bis-Tris gel (Invitrogen) was loaded with 15μL of each sample in duplicate and run in MOPS buffer at 200V for 50 minutes. The gels were then transferred onto a 0.45 μm nitrocellulose membrane (Bio-Rad) using a genie transfer apparatus (Idea Scientific) at 12V for 90 minutes. The membrane was incubated in blocking buffer (5% milk in TBST) at room temperature for 1 hour before cutting the membrane in half and incubating with a 1:1000 dilution of either primary anti-HA (Cell Signaling 3724T) or primary anti-GFP (Cell Signaling 29565) in blocking buffer and incubated at 4°C overnight. The following day membranes were washed 3x with 10mL of blocking buffer followed by a 1-hour incubation in a 1:20,000 dilution of IRDye 800CW secondary Ab (LI-COR 926–32213) in blocking buffer. The membrane was washed 3x in TBST and imaged on an Odyssey imaging system (LI-COR).

### RESOURCE AVAILABILITY

#### Lead Contact

Requests for further information and resources should be directed to and will be fulfilled by the lead contact, Evan Eichler (ee3@uw.edu).

#### Materials Availability

DNA sequence of plasmids generated in this study for NOTCH2NL protein expression have been deposited to Github (https://github.com/tdreal/NOTCH2NL-0325/tree/main) and Zenodo (https://zenodo.org/records/15022214).

#### Data and Code Availability

PacBio HiFi Fiber-seq and Kinnex full-length Iso-Seq from HG02630 neurospheres generated for this study have been made available on NCBI with the BioProject ID: PRJNA1236375. Original western blot images are deposited to Github (https://github.com/tdreal/NOTCH2NL-0325/tree/main) and Zenodo (https://zenodo.org/records/15022214). There is no original code reported by this study. Any additional information required to reanalyze the data reported in this work paper is available from the lead contact upon request.

## Supplementary Material

Supplement 1

## Figures and Tables

**Table 1.** Greater *NOTCH2NL* region pairwise homology matrix in T2T-CHM13

	NOTCH2	NOTCH2NLR	NOTCH2NLA	NOTCH2NLB	NOTCH2NLC

*NOTCH2*	x	115,690	326,052	556,583	118,987
*NOTCH2NLR*	99.7	x	342,476	328,624	217,296
*NOTCH2NLA*	99.3	99.3	x	416,088	182,193
*NOTCH2NLB*	99.3	99.3	99.7	x	265,598
*NOTCH2NLC*	99.2	99.0	99.1	99.2	x
