# Supplementary material for: Genetic diversity and regulatory features of human-specific NOTCH2NL duplications
Source: bioRxiv. 2025 Mar 17:2025.03.14.643395. Preprint. [Version 2] doi: 10.1101/2025.03.14.643395 (PMC11956922; doi:10.1101/2025.03.14.643395)
Supplement: Supplement 1 [file media-1.docx]

**SUPPLEMENTARY MATERIAL**

| **Supplementary Table 1. Structural variants of *NOTCH2NL* paralogs in T2T-CHM13** | | | | | | |
| --- | --- | --- | --- | --- | --- | --- |
| query paralog | ref paralog | query start | query end | mapq | structural variant size | in. (I) / del (D) |
| NOTCH2 | NOTCH2NLC | 119846347 | 119900735 | 60 | 54389 | I |
| NOTCH2 | NOTCH2NLC | 119582683 | 119582682 | 60 | 737 | D |
| NOTCH2 | NOTCH2NLC | 119541422 | 119547699 | 60 | 6278 | I |
| NOTCH2 | NOTCH2NLC | 119577460 | 119579275 | 60 | 1816 | I |
| NOTCH2 | NOTCH2NLB | 119542857 | 119555494 | 60 | 12638 | I |
| NOTCH2 | NOTCH2NLB | 119814488 | 119818620 | 60 | 4133 | I |
| NOTCH2 | NOTCH2NLB | 119461254 | 119461253 | 60 | 5629 | D |
| NOTCH2 | NOTCH2NLB | 119461388 | 119461387 | 60 | 4741 | D |
| NOTCH2 | NOTCH2NLB | 119462558 | 119462557 | 60 | 996 | D |
| NOTCH2 | NOTCH2NLB | 119677199 | 119677198 | 60 | 748 | D |
| NOTCH2 | NOTCH2NLB | 119857611 | 119857610 | 60 | 3875 | D |
| NOTCH2 | NOTCH2NLB | 119917835 | 119917834 | 60 | 1358 | D |
| NOTCH2 | NOTCH2NLB | 120135864 | 120137057 | 60 | 1194 | I |
| NOTCH2 | NOTCH2NLB | 120134461 | 120135831 | 60 | 1371 | I |
| NOTCH2 | NOTCH2NLA | 119814488 | 119818620 | 60 | 4133 | I |
| NOTCH2 | NOTCH2NLA | 119677199 | 119677198 | 60 | 748 | D |
| NOTCH2 | NOTCH2NLA | 119857611 | 119857610 | 60 | 3852 | D |
| NOTCH2 | NOTCH2NLA | 119917825 | 119917824 | 60 | 1360 | D |
| NOTCH2 | NOTCH2NLA | 119542836 | 119555473 | 60 | 12638 | I |
| NOTCH2 | NOTCH2NLA | 119462604 | 119462603 | 60 | 996 | D |
| NOTCH2 | NOTCH2NLA | 119461389 | 119461388 | 60 | 4741 | D |
| NOTCH2 | NOTCH2NLA | 119461253 | 119461252 | 60 | 5629 | D |
| NOTCH2 | NOTCH2NLA | 120134365 | 120134947 | 31 | 583 | I |
| NOTCH2 | NOTCH2NLA | 120135063 | 120135832 | 31 | 770 | I |
| NOTCH2 | NOTCH2NLA | 120135863 | 120137056 | 31 | 1194 | I |
| NOTCH2NLA | NOTCH2NLC | 145263860 | 145265217 | 60 | 1358 | I |
| NOTCH2NLA | NOTCH2NLC | 145426571 | 145428098 | 60 | 1528 | I |
| NOTCH2NLA | NOTCH2NLC | 145431274 | 145434427 | 60 | 3154 | I |
| NOTCH2NLA | NOTCH2NLC | 144857035 | 144858873 | 60 | 1839 | I |
| NOTCH2NLA | NOTCH2NLB | 145373282 | 145374823 | 60 | 1542 | I |
| NOTCH2NLA | NOTCH2NLB | 145426775 | 145428316 | 60 | 1542 | I |
| NOTCH2NLA | NOTCH2NLB | 145357941 | 145357940 | 60 | 6296 | D |
| NOTCH2NLA | NOTCH2NLB | 145452444 | 145455266 | 60 | 2823 | I |
| NOTCH2NLB | NOTCH2NLC | 146024204 | 146083108 | 60 | 58905 | I |
| NOTCH2NLB | NOTCH2NLC | 146100183 | 146101538 | 60 | 1356 | I |
| NOTCH2NLB | NOTCH2NLC | 146196656 | 146202946 | 60 | 6291 | I |
| NOTCH2NLB | NOTCH2NLC | 146270637 | 146273780 | 60 | 3144 | I |
| NOTCH2NLB | NOTCH2NLC | 146282918 | 146295060 | 60 | 12143 | I |
| NOTCH2NLB | NOTCH2NLC | 146295069 | 146303567 | 60 | 8499 | I |
| NOTCH2NLB | NOTCH2NLC | 146215843 | 146215842 | 60 | 1549 | D |
| NOTCH2NLB | NOTCH2NLC | 146461653 | 146461652 | 60 | 2066 | D |
| NOTCH2NLB | NOTCH2NLC | 145759226 | 145761064 | 60 | 1839 | I |
| NOTCH2NLC | NOTCH2NLR | 148580984 | 148584007 | 60 | 3024 | I |
| NOTCH2NLC | NOTCH2NLR | 148623116 | 148626328 | 60 | 3213 | I |
| NOTCH2NLC | NOTCH2NLR | 148627877 | 148631089 | 60 | 3213 | I |
| NOTCH2NLC | NOTCH2NLR | 148635700 | 148678456 | 60 | 42757 | I |
| NOTCH2NLC | NOTCH2NLR | 148608665 | 148608664 | 60 | 6274 | D |
| NOTCH2NLC | NOTCH2NLR | 148687503 | 148687502 | 60 | 11467 | D |
| NOTCH2NLC | NOTCH2NLR | 148687511 | 148687510 | 60 | 8199 | D |
| NOTCH2NLC | NOTCH2NLR | 148867048 | 148870884 | 60 | 3837 | I |
| NOTCH2NLC | NOTCH2NLR | 148443146 | 148443145 | 60 | 11467 | D |
| NOTCH2NLC | NOTCH2NLR | 148443154 | 148443153 | 60 | 8199 | D |
| NOTCH2NLC | NOTCH2NLR | 148335672 | 148335671 | 60 | 1816 | D |
| NOTCH2NLR | NOTCH2NLB | 120452239 | 120464876 | 60 | 12638 | I |
| NOTCH2NLR | NOTCH2NLB | 120546480 | 120546479 | 60 | 5629 | D |
| NOTCH2NLR | NOTCH2NLB | 120546346 | 120546345 | 60 | 4741 | D |
| NOTCH2NLR | NOTCH2NLB | 120545176 | 120545175 | 60 | 996 | D |
| NOTCH2NLR | NOTCH2NLB | 120330535 | 120330534 | 60 | 748 | D |
| NOTCH2NLR | NOTCH2NLB | 120730144 | 120730143 | 60 | 1356 | D |
| NOTCH2NLR | NOTCH2NLB | 120798010 | 120798009 | 60 | 3025 | D |
| NOTCH2NLR | NOTCH2NLB | 120841724 | 120841723 | 60 | 3184 | D |
| NOTCH2NLR | NOTCH2NLB | 120843404 | 120843403 | 60 | 3172 | D |
| NOTCH2NLR | NOTCH2NLB | 120845053 | 120845052 | 60 | 3202 | D |
| NOTCH2NLR | NOTCH2NLB | 120849542 | 120849541 | 60 | 40814 | D |
| NOTCH2NLR | NOTCH2NLB | 120861687 | 120861686 | 60 | 654 | D |
| NOTCH2NLR | NOTCH2NLB | 120594342 | 120594341 | 60 | 748 | D |
| NOTCH2NLR | NOTCH2NLA | 120452260 | 120464897 | 60 | 12638 | I |
| NOTCH2NLR | NOTCH2NLA | 120545130 | 120545129 | 60 | 996 | D |
| NOTCH2NLR | NOTCH2NLA | 120546345 | 120546344 | 60 | 4741 | D |
| NOTCH2NLR | NOTCH2NLA | 120546481 | 120546480 | 60 | 5629 | D |
| NOTCH2NLR | NOTCH2NLA | 120594342 | 120594341 | 60 | 748 | D |
| NOTCH2NLR | NOTCH2NLA | 120820315 | 120826597 | 60 | 6283 | I |
| NOTCH2NLR | NOTCH2NLA | 120730144 | 120730143 | 60 | 1358 | D |
| NOTCH2NLR | NOTCH2NLA | 120798010 | 120798009 | 60 | 3025 | D |
| NOTCH2NLR | NOTCH2NLA | 120843404 | 120843403 | 60 | 3180 | D |
| NOTCH2NLR | NOTCH2NLA | 120844944 | 120844943 | 60 | 3168 | D |
| NOTCH2NLR | NOTCH2NLA | 120330535 | 120330534 | 60 | 748 | D |
| NOTCH2NLR | NOTCH2 | 120798010 | 120798009 | 60 | 3028 | D |
| NOTCH2NLR | NOTCH2 | 120838292 | 120846100 | 60 | 7809 | I |

| **Supplementary Table 2. Additional structural variation between human and NHA** | | | | | | | | |
| --- | --- | --- | --- | --- | --- | --- | --- | --- |
| Variant information | | | NOTCH2 mapping (primary hap) | | | INV BP mapping (T2T-space; kbp) | | |
| Species | Variants | Count | Bases (kbp) | Count | Bases (kbp) | Count | Bases (kbp) | p-value |
| PPY | PAV-DEL | 816 | 752.8 | 3 | 0.7 | 71 | 99.5 | 1.000 |
| PPY | PAV-INS | 829 | 1025.1 | 6 | 9.1 | 82 | 140.0 | 1.000 |
| PPY | Syri/PAV-INV | 6 | 930.4 | - | - | - | - | - |
| PPY | Sedef-SD (h1/h2) | 98/97 | 2588.9/2588.9 | 1 | 10.6 | - | - | - |
| PAB | PAV-DEL | 810 | 751.7 | 3 | 0.7 | 72 | 106.1 | 1.000 |
| PAB | PAV-INS | 812 | 1016.1 | 5 | 9.0 | 74 | 123.7 | 1.000 |
| PAB | Syri/PAV-INV | 4 | 1338.3 | - | - | - | - | - |
| PAB | Sedef-SD (h1/h2) | 97/95 | 2611.4/2576.7 | 1 | 10.3 | - | - | - |
| PTR | PAV-DEL | 343 | 341.3 | 3 | 5.2 | 29 | 69.3 | 0.987 |
| PTR | PAV-INS | 312 | 1146.2 | 2 | 604.9 | 40 | 883.4 | **<0.001*** |
| PTR | Syri/PAV-INV | 3 | 8017.1 | - | - | - | - | - |
| PTR | Sedef-SD (h1/h2) | 109/111 | 6565.9/6582.8 | 10 | 3660.8 | - | - | - |
| GGO | PAV-DEL | 475 | 591.1 | 5 | 11.1 | 36 | 141.7 | 0.832 |
| GGO | PAV-INS | 422 | 1010.9 | 0 | 0.0 | 35 | 223.8 | **0.034*** |
| GGO | Syri/PAV-INV | 6 | 8885.6 | - | - | - | - | - |
| GGO | Sedef-SD (pri/alt) | 114/118 | 7103.1/7049.3 | 6 | 3043.9 | - | - | - |
| PPA | PAV-DEL | 353 | 359.9 | 3 | 5.2 | 19 | 51.0 | 0.999 |
| PPA | PAV-INS | 308 | 537.7 | 1 | 3.0 | 25 | 262.5 | **<0.001*** |
| PPA | Syri/PAV-INV | 6 | 9691.5 | - | - | - | - | - |
| PPA | Sedef-SD (pri/alt) | 110/111 | 6459.5/6059.9 | 10 | 3532.2 | - | - | - |
| HSA | Sedef-SD | 91 | 8315.6 | 3 | 3988.8 | 30 | 1918.8 | **<0.001*** |
|  |  |  | **Average** | 3.9 | 931.0 | 46.6 | 365.4 |  |

| **Supplementary Table 3. NHA *NOTCH2NL* FLNC characterization** | | | | | | | | | | | |
| --- | --- | --- | --- | --- | --- | --- | --- | --- | --- | --- | --- |
| Name | Iso-Seq transcript | Chr | Start | End | Strand | # of exons | Predicted AA Lengths | Long-read transcript support (NEC & testis) | Intergenic distance N2NL-NBPF (bp) | Species | Comments |
| PTR-NOTCH2 | NOTCH2 | chr1 | 117131242 | 117288384 | - | -- | 2432 | -- | -- | PTR/PPA/GGO/HSA | -- |
| PTR-NOTCH2NL-1 | NOTCH2NLR | chr1 | 106135723 | 106156373 | + | 4 | 246 | 26 | 11,622 | PPA/PTR | ORF has exons 1, 3-5 of NOTCH2NLR |
|  | Fusion NOTCH2NL-NBPF | chr1 | 106135792 | 106185074 |  | 20 | 851 | 69 |  |  | ORF has exons 1, 3, 4 of NOTCH2NLR & NBPF exons |
| PTR-NOTCH2NL-2 | Fusion MAGI3-NOTCH2NL | chr1 | 106365562 | 106516651 | + | 4 | 324 | 6 | 11,398 | PPA/PTR | ORF has MAGI3 exon 1 & exons 3-5 of NOTCH2NLR |
| PTR-NOTCH2NL-3 | NOTCH2NLR | chr1 | 108282469 | 108322382 | + | 4 | 235 | 16 | 11,838 | PPA/PTR/GGO | ORF has exons 2-5 of NOTCH2NLR |
|  | Fusion NOTCH2NL-NBPF | chr1 | 108282319 | 108349249 |  | 13 | 672 | 11 |  |  | ORF has exons 2, 3, 4 of NOTCH2NLR & NBPF exons |
|  | Fusion PDE4DIP-NOTCH2NL-NBPF | chr1 | 108214852 | 108349197 |  | -- | -- | 53 |  |  | No ORF goes through the whole transcript |
| PTR-NOTCH2NL-4 | Fusion MAGI3-NOTCH2NL | chr1 | 109104609 | 109227579 | - | 13 | 845 | 12 | 11,410 | PPA/PTR | ORF has exon 1 of NOTCH2NLR, 9 MAGI3, and 3-5 exons of NOTCH2NLR |
|  | Fusion MAGI3-NOTCH2NL-NBPF | chr1 | 109078294 | 109227828 |  | 17 | 1,411 | 53 |  |  | ORF has exon 1 of NOTCH2NLR, 9 MAGI3, NOTCH2NL exons 3, 4, and NBPF exons |
| PTR-NOTCH2NL-5 | NOTCH2NLR | chr1 | 109873983 | 109910441 | - | 3 | 146 | 9 | 11,295 | PPA/PTR | ORF has exons 2, 3 of NOTCH2NLR & partial exon 4 (TRUNCATION) - transcript goes through until NOTCH2NLR-like exon 5 |
|  | Fusion NOTCH2NL-NBPF | chr1 | 109846910 | 109910766 |  | 3 | 146 | 12 |  |  | ORF has exons 2, 3 of NOTCH2NLR & partial exon 4 (TRUNCATION) - transcript goes through to NBPF exons |
|  | Fusion TXNIP-NOTCH2NL-NBPF | chr1 | 109846504 | 109953395 |  | 4 | 230 | 62 |  |  | ORF has exon 1 of TXNIP, exons 2,3 of NOTCH2NLR, and partial exon 4 (TRUNCATION) - transcript goes through until NOTCH2NLR-like exon 5 |
| PTR-NOTCH2NL-6 | NOTCH2NLR | chr1 | 110315936 | 110336372 | + | 4 | 243 | 11 | 11,504 | PPA/PTR/GGO | ORF has exon 1, 3-5 of NOTCH2NLR |
|  | Fusion NOTCH2NL-NBPF | chr1 | 110313408 | 110368080 |  | 20 | 856 | 22 |  |  | ORF that has exons 1, 3, 4 of NOTCH2NLR & NBPF exons |
|  | Fusion SORT1-LRIG2-NOTCH2NL-NBPF | chr1 | 110225344 | 110368328 |  | -- | -- | 14 |  |  | No ORF goes through the whole transcript |
| PTR-NOTCH2NL-7 | Fusion with MAGI3 | chr1 | 117544406 | 117696496 | - | 4 | 324 | 7 | 11,693 | PPA/PTR | ORF has exon 1 of MAGI3 & exons 3-5 of NOTCH2NLR |
| PTR-NOTCH2NL-8 | NOTCH2NLR | chr1 | 118044677 | 118065257 | - | 4 | 246 | 9 | 11,295 | PTR | ORF has exons 1, 3-5 of NOTCH2NLR |
|  | Fusion NOTCH2NL-NBPF | chr1 | 117960416 | 118065110 |  | 58 | 3033 | 16 |  |  | ORF has exons 1, 3, 4 of NOTCH2NLR & NBPF exons |
|  | Fusion LRIG2-NOTCH2NL | chr1 | 118044029 | 118124464 |  | -- | -- | 5 |  |  | No ORF goes through the whole transcript |
|  | Fusion LRIG2-NOTCH2NL-NBPF | chr1 | 117960416 | 118125899 |  | -- | -- | 4 |  |  | No ORF goes through the whole transcript |
| PTR-NOTCH2NL-9 | NOTCH2NLR | chr1 | 121665898 | 121686392 | + | 4 | 245 | 11 | 11,486 | PPA/PTR | ORF has exons 1, 3-5 of NOTCH2NLR |
|  | Fusion NOTCH2NL-NBPF | chr1 | 121665622 | 121801595 |  | 88 | 4694 | 48 |  |  | ORF has exons 1, 3, 4 of NOTCH2NLR & NBPF exons |
| PPA-NOTCH2 | NOTCH2 | chr1 | 112555608 | 112716322 | - | -- | 2610 | -- | -- | PTR/PPA/GGO/HSA | -- |
| PPA-NOTCH2NL-1 | NOTCH2NLR | chr1 | 101669793 | 101703165 | - | 4 | 235 | 46 | 11,607 | PPA/PTR/GGO | ORF has exons 2-5 of NOTCH2NLR human |
|  | Fusion NOTCH2NL-NBPF | chr1 | 101642931 | 101703233 |  | 17 | 656 | 132 |  |  | ORF has exons 2, 3, 4 of NOTCH2NLR & NBPF exons |
|  | Fusion PDE4DIP-NOTCH2NL-NBPF | chr1 | 101641810 | 101777882 |  | -- | -- | 155 |  |  | No ORF goes through the whole transcript. |
| PPA-NOTCH2NL-2 | Fusion MAGI3-NOTCH2NL | chr1 | 103485748 | 103636324 | - | 4 | 237 | 6 | 11,837 | PPA/PTR | ORF has exon 1 of MAGI3 & exons 3-5 of NOTCH2NLR |
| PPA-NOTCH2NL-3 | NOTCH2NLR | chr1 | 103841187 | 103861631 | + | 4 | 246 | 11 | 11,672 | PPA/PTR | ORF has exons 1, 3-5 of NOTCH2NLR |
|  | Fusion NOTCH2NL-NBPF | chr1 | 103815726 | 103861620 |  | 15 | 757 | 98 |  |  | ORF has exons 1, 3, 4 of NOTCH2NLR & NBPF exons |
| PPA-NOTCH2NL-4 | Fusion MAGI3-NOTCH2NL | chr1 | 104697226 | 104820526 |  | 13 | 845 | 201 | -- | PPA/PTR | ORF has exon 1 of NOTCH2NLR, 9 MAGI3, & 3-5 exons of NOTCH2NLR |
| PPA-NOTCH2NL-5 | NOTCH2NLR | chr1 | 105315877 | 105349528 | + | 4 | 235 | 11 | 11,690 | PPA/PTR | ORF has exons 2-5 of NOTCH2NLR |
|  | Fusion NOTCH2NL-NBPF | chr1 | 105288382 | 105349479 |  | 15 | 743 | 69 |  |  | ORF has exons 2, 3, 4 of NOTCH2NLR & NBPF exons |
|  | Fusion TXNIP-NOTCH2NL-NBPF | chr1 | 105286533 | 105389706 |  | 5 | 319 | 21 |  |  | ORF has exon 1 of TXNIP & exons 2, 3-5 of NOTCH2NLR |
| PPA-NOTCH2NL-6 | NOTCH2NLR | chr1 | 105752206 | 105772648 | - | 4 | 246 | 7 | 11,773 | PPA/PTR | ORF has exons 1, 3-5 of NOTCH2NLR |
|  | Fusion NOTCH2NL-NBPF | chr1 | 105752087 | 105795054 |  | 14 | 682 | 81 |  |  | ORF has exons 1, 3, 4 of NOTCH2NLR & NBPF exons |
| PPA-NOTCH2NL-7 | NOTCH2NLR | chr1 | 112968284 | 112977175 | - | 4 | 246 | 0 | 11,194 | PPA | ORF has exons 3-5 of NOTCH2NLR |
| PPA-NOTCH2NL-8 | Fusion MAGI3-NOTCH2NL | chr1 | 113432163 | 113583978 | - | 4 | 324 | 12 | 11,233 | PPA/PTR | ORF has exon 1 of MAGI3 & exons 3-5 of NOTCH2NLR |
| PPA-NOTCH2NL-9 | NOTCH2NLR | chr1 | 113892494 | 113913027 | - | 4 | 246 | 56 | 11,785 | PPA | ORF has exons 1, 3-5 of NOTCH2NLR |
| PPA-NOTCH2NL-10 | NOTCH2NLR | chr1 | 117521012 | 117542196 | + | 4 | 246 | 12 | 11,731 | PPA/PTR/GGO | ORF has exons 1, 3-5 of NOTCH2NLR |
|  | Fusion NOTCH2NL-NBPF | chr1 | 117520465 | 117596479 |  | 50 | 2399 | 35 |  |  | ORF has exons 1, 3, 4 of NOTCH2NLR & NBPF exons |
|  | Fusion SORT1-LRIG2-NOTCH2NL-NBPF | chr1 | 117431222 | 117596326 |  | -- | -- | 4 |  |  | No ORF goes through the whole transcript |
| GGO-NOTCH2 | NOTCH2 | chr1 | 124746752 | 124900741 | - | -- | 2611 | -- | -- | PTR/PPA/GGO/HSA | -- |
| GGO-NOTCH2NL-1 | NOTCH2NLR | chr1 | 116765261 | 116796179 | + | -- | -- | 2 | 11,618 | PPA/PTR/GGO | Has exon 3-5 of NOTCH2NLR but no exon 1/2 and no M start |
|  | Fusion NOTCH2NL-NBPF | chr1 | 116765192 | 116847395 |  | -- | -- | 13 |  |  | Has exon 3-4 of NOTCH2NLR & NBPF exons but no exon 1/2 & no M start |
|  | Fusion SORT1-LRIG2-NOTCH2NL-NBPF | chr1 | 116693440 | 116846869 |  | -- | -- | 6 |  |  | No ORF goes through the whole transcript. |
| GGO-NOTCH2NL-2 | NOTCH2NLR | chr1 | 117331132 | 117364496 | - | 4 | 235 | 5 | 11,825 | PPA/PTR/GGO | ORF has exons 2-5 of NOTCH2NLR |
|  | Fusion NOTCH2NL-NBPF | chr1 | 117294017 | 117373312 |  | 24 | 1114 | 11 |  |  | ORF has exons 2-4 of NOTCH2NLR & NBPF exons |
|  | Fusion PDE4DIP-NOTCH2NL-NBPF | chr1 | 117294017 | 117447550 |  | -- | -- | 16 |  |  | No ORF goes through the whole transcript. |
| GGO-NOTCH2NL-3 | Fusion LRIG2-NOTCH2NL-NBPF | chr1 | 125591450 | 125661487 | - | -- | 1662 | 5 | 5385 | GGO | Has exons of LRIG2, exon 3 of NOTCH2NLR, & NBPF exons but no M start |
|  | Fusion NOTCH2NL-NBPF | chr1 | 125591876 | 125635112 |  | -- | -- | 8 |  |  | Has exon 3 of NOTCH2NLR and NBPF exons but no exon 1/2 and no M start |
| GGO-NOTCH2NL-4 | Fusion LRIG2-NOTCH2NL-NBPF | chr1 | 129364798 | 129435830 | + | -- | -- | 19 | 5,608 | GGO | Has 2 LRIG2 exons, exon 3 of NOTCH2NLR, & NBPF exons but early stop in exon 2 |
|  | Fusion NOTCH2NL-NBPF | chr1 | 129389184 | 129435600 |  | -- | -- | 2 |  |  | Has 3-4 of NOTCH2NLR & NBPF exons but no exon 1/2 and no M start |
| GGO-NOTCH2NL-5 | Fusion MAGI3-NOTCH2NL-NBPF | chr1 | 129726316 | 129995372 | + | -- | 1487 | 2 | 5,588 | GGO | Has exons of MAGI3, exon 3 of NOTCH2NLR, & then NBPF exons but no M start |
|  | Fusion NOTCH2NL-NBPF | chr1 | 129964606 | 129995476 |  | -- | -- | 6 |  |  | Has 3-4 of NOTCH2NLR & NBPF exons but no exon 1/2 and no M start |
| GGO-NOTCH2NL-6 | Fusion NOTCH2NL-NBPF | chr1 | 130013152 | 130080198 | - | 13 | 783 | 72 | 39,879 | GGO | ORF has exon 1 of NOTCH2NLR & NBPF exons |
| GGO-NOTCH2NL-7 | NOTCH2NLR | chr1 | 130265287 | 130274296 | - | -- | -- | 14 | 11,779 | GGO | Has 3-5 of NOTCH2NLR but no exon 1/2 and no M start |
|  | Fusion NOTCH2NL-NBPF | chr1 | 130222211 | 130280603 |  | -- | -- | 15 |  |  | Has 3-4 of NOTCH2NLR & NBPF exons but no exon 1/2 and no M start |
|  | Fusion BRD9-NOTCH2NL | chr1 | 130264240 | 130305169 |  | -- | -- | 8 |  |  | No ORF that goes through the whole transcript. |
|  | Fusion BRD9-NOTCH2NL-NBPF | chr1 | 130221951 | 130304952 |  | -- | -- | 4 |  |  | No ORF that goes through the whole transcript. |
| HSA-NOTCH2 | NOTCH2 | chr1 | 119924936 | 120082923 | - | -- | 2471 | -- | -- | PTR/PPA/GGO/HSA | -- |
| HSA-NOTCH2NLA | NOTCH2NLA | chr1 | 145272197 | 145345902 | - | 5 | 236 | -- | 11,336 | HSA |  |
| HSA-NOTCH2NLB | NOTCH2NLB | chr1 | 146108509 | 146181500 | + | 5 | 249 | -- | 11,375 | HSA |  |
| HSA-NOTCH2NLC | NOTCH2NLC | chr1 | 148535272 | 148596912 | + | 5 | 236 | -- | 11,726 | HSA |  |
| HSA-NOTCH2NLR | NOTCH2NLR | chr1 | 120737165 | 120807117 | + | 5 | 274 | -- | 11,635 | HSA |  |
|  | ^a^Fusion NOTCH2NL-NBPF | chr1 | 120737168 | 120851627 |  | 36 | 1673 | -- |  |  |  |

^a^Fusion exists in all human *NOTCH2NL* but only *NOTCH2NLR* fusion is used for comparison.

| **Supplementary Table 4. Population origin of HPRC haplotypes resolved^a^ across the *NOTCH2NL* locus** | | | |
| --- | --- | --- | --- |
| superpopulation | superpopulation total | population | population total |
| AFR | 34 | African Caribbean in Barbados | 11 |
|  |  | Gambian in Western Divisions - Mandinka | 15 |
|  |  | Mende in Sierra Leone | 6 |
|  |  | Yoruba in Ibadan, Nigeria | 2 |
|  |  | African Ancestry in Southwest USA | 2 |
|  |  | Maasai in Kinyawa, Kenya | 1 |
| AMR | 23 | Puerto Rican in Puerto Rico | 14 |
|  |  | Colombian in Medellin, Colombia | 2 |
|  |  | Peruvian in Lima, Peru | 7 |
| EAS | 7 | Kinh in Ho Chi Minh City, Vietnam | 1 |
|  |  | Southern Han Chinese in Hu Nan Province, China | 5 |
|  |  | Chinese Ancestry in USA | 1 |
| EUR | 1 | Ashkenazim Jewish | 1 |
| SAS | 1 | Punjabi in Lahore, Pakistan | 1 |

^a^This includes haplotypes that are not completely assembled across the centromere but have no gaps or collapses between *NOTCH2* and *NOTCH2NLR*, and *NOTCH2NLA/B/C*, respectively.

| **Supplementary Table 5. *NOTCH2NL*/*GFP* gBlocks for measuring protein expression** | |
| --- | --- |
| **Sequence name** | **5' – sequence – 3'** |
| HA-NOTCH2tv-IRES-NheI-FseI gBlock | GGTCTAGAGCTAGCGAATTCGCCGGTGCCACCATGTACCCATACGATGTTCCAGATTACGCTCCCGCCCTGCGCCCCGCTCTGCTGTGGGCGCTGCTGGCGCTCTGGCTGTGCTGCGCGGCCCCCGCGCATGCATTGCAGTGTCGAGATGGCTATGAACCCTGTGTAAATGAAGGAATGTGTGTTACCTACCACAATGGCACAGGATACTGCAAATGTCCAGAAGGCTTCTTGGGGGAATATTGTCAACATCGAGACCCCTGTGAGAAGAACCGCTGCCAGAATGGTGGGACTTGTGTGGCCCAGGCCATGCTGGGGAAAGCCACGTGCCGATGTGCCTCAGGGTTTACAGGAGAGGACTGCCAGTACTCAACATCTCATCCATGCTTTGTGTCTCGACCCTGCCTGAATGGCGGCACATGCCATATGCTCAGCCGGGATACCTATGAGTGCACCTGTCAAGTCGGGTTTACAGGTAAGGAGTGCCAATGGACGGATGCCTGCCTGTCTCATCCCTGTGCAAATGGAAGTACCTGTACCACTGTGGCCAACCAGTTCTCCTGCAAATGCCTCACAGGCTTCACAGGGCAGAAATGTGAGACTGATGTCAATGAGTGTGACATTCCAGGACACTGCCAGCATGGTGGAACCTGCCTCAACCTGCCTGGTTCCTACCAGTGCCAGTGCCCTCAGGGCTTCACAGGCCAGTACTGTGACAGCCTGTATGTGCCCTGTGCACCCTCACCTTGTGTCAATGGAGGTACCTGTCGGCAGACTGGTGACTTCACTTTTGAGTGCAACTGCCTTCCAGAAACAGTGAGAAATAAGAGGAACAGAGCTCTGGGAAAGGGACAGGCAAGTCTGGAATGGAAAAGAACATGAGTCGACCGCTTGGAATAAGGCCGGTGTGCGTTTGTCTATATGTTATTTTCCACCATATTGCCGTCTTTTGGCAATGTGAGGGCCCGGAAACCTGGCCCTGTCTTCTTGACGAGCATTCCTAGGGGTCTTTCCCCTCTCGCCAAAGGAATGCAAGGTCTGTTGAATGTCGTGAAGGAAGCAGTTCCTCTGGAAGCTTCTTGAAGACAAACAACGTCTGTAGCGACCCTTTGCAGGCAGCGGAACCCCCCACCTGGCGACAGGTGCCTCTGCGGCCAAAAGCCACGTGTATAAGATACACCTGCAAAGGCGGCACAACCCCAGTGCCACGTTGTGAGTTGGATAGTTGTGGAAAGAGTCAAATGGCTCTCCTCAAGCGTATTCAACAAGGGGCTGAAGGATGCCCAGAAGGTACCCCATTGTATGGGATCTGATCTGGGGCCTCGGTGCACATGCTTTACATGTGTTTAGTCGAGGTTAAAAAAACGTCTAGGCCCCCCGAACCACGGGGACGTGGTTTTCCTTTGAAAAACACGATGATAAGATCTGCGATCTAAGTAAGCTTGGCATTCCGGTACTGTTGGTAAAGCCACCATGGAATCCGGCCGGCCCGAATTCGGC |
| HA-NOTCH2NLB pEF1A Gibson gBlock | GATGTTCCAGATTACGCTTGTCGAGATGGCTATGAACCCTGTGTAAATGAAGGAATGTGTGTTACCTACCACAATGGCACAGGATACTGCAAATGTCCAGAAGGCTTCTTGGGGGAATATTGTCAACATCGAGACCCCTGTGAGAAGAACCGCTGCCAGAATGGTGGGACTTGTGTGGCCCAGGCCATGCTGGGGAAAGCCACGTGCCGATGTGCCTCAGGGTTTACAGGAGAGGACTGCCAGTACTCGACATCTCATCCATGCTTTGTGTCTCGACCTTGCCTGAATGGCGGCACATGCCATATGCTCAGCCGGGATACCTATGAGTGCACCTGTCAGGTCGGGTTTACAGGTAAGGAGTGCCAATGGACCGATGCCTGCCTGTCTCATCCCTGTGCAAATGGAAGTACCTGTACCACTGTGGCCAACCAGTTCTCCTGCAAATGCCTCACAGGCTTCACAGGGCAGAAGTGTGAGACTGATGTCAATGAGTGTGACATTCCAGGACACTGCCAGCATGGTGGCATCTGCCTCAACCTGCCTGGTTCCTACCAGTGCCAGTGCCTTCAGGGCTTCACAGGCCAGTACTGTGACAGCCTGTATGTGCCCTGTGCACCCTCGCCTTGTGTCAATGGAGGCACCTGTCGGCAGACTGGTGACTTCACTTTTGAGTGCAACTGCCTTCCAGAAACAGTGAGAAGAGGAACAGAGCTCTGGGAAAGAGACAGGGAAGTCTGGAATGGAAAAGAACACGATGAGAATTAGGTCGACCGCTTGGAATAA |
| HA-NOTCH2NLR pEF1A Gibson gBlock | GATGTTCCAGATTACGCTCCCGCCCTGCGTCCCGCTCTGCTGTGGGCGCTGCTGGCGCTCTGGCTGTGCTGGGCGGCCCCCGCGCATGCATTGCAGTGTCGAGATGGCTATGAACCCTGTGTAAATAAAGGAATGTGTGTTACCTACCACAGTGGCACAGGATACTGCAAATGTCCAGAAGGCTTCTTGGGGGAATATTGTCAACATCGAGACCCCTGTGAGAAGAACCGCTGCCAGAATGGTGGGACTTGTGTGGCCCAGGCCATGCTGGGGAAAGCCACGTGCCGGTGTGCCTCAGGGTTTACAGGAGAGGACTGCCAGTACTCGACACCTCATCCATGCTTTGTGTCTCGACCTTGCCTGAATGGCGGCACATGCCATATGCTCAGCCGGGATACCTATGAGTGCACCTGTCAAGTCGGGTTTACAGGTAAGGAGTGCCAATGGACCGATGCCTGCCTGTCTCATCTCTGTGCAAATGGAAGTACCTGTACCACTGTGGCCAACCAGTTCTCCTGCAAATGCCTCACAGGCTTCACAGGGCAGAAGTGTGAGACTGATGTCAATGAGTGTGACATTCCAGGACACTGCCAGCATGGTGGCACCTGCCTCAACCTGCCTGGTTCCTACCAGTGCCAGTGCCTTCAGGGCTTCACAGGCCAGTACTGTGACAGACTGTATGTGCCCTGTGCACACTCGCCTTGTGTCAATGGAGGCACCTGTCGGCAGACTGGTGACTTCACTTTTGAGTGCAACTGCCTTCCAGAAACAGTGAGAAATAAGAGGAACAGAGCTCTGGGAAAGAGACAGGCAAGTCTGGAATGGAAAAGAACACGATGAGTCGACCGCTTGGAATAA |


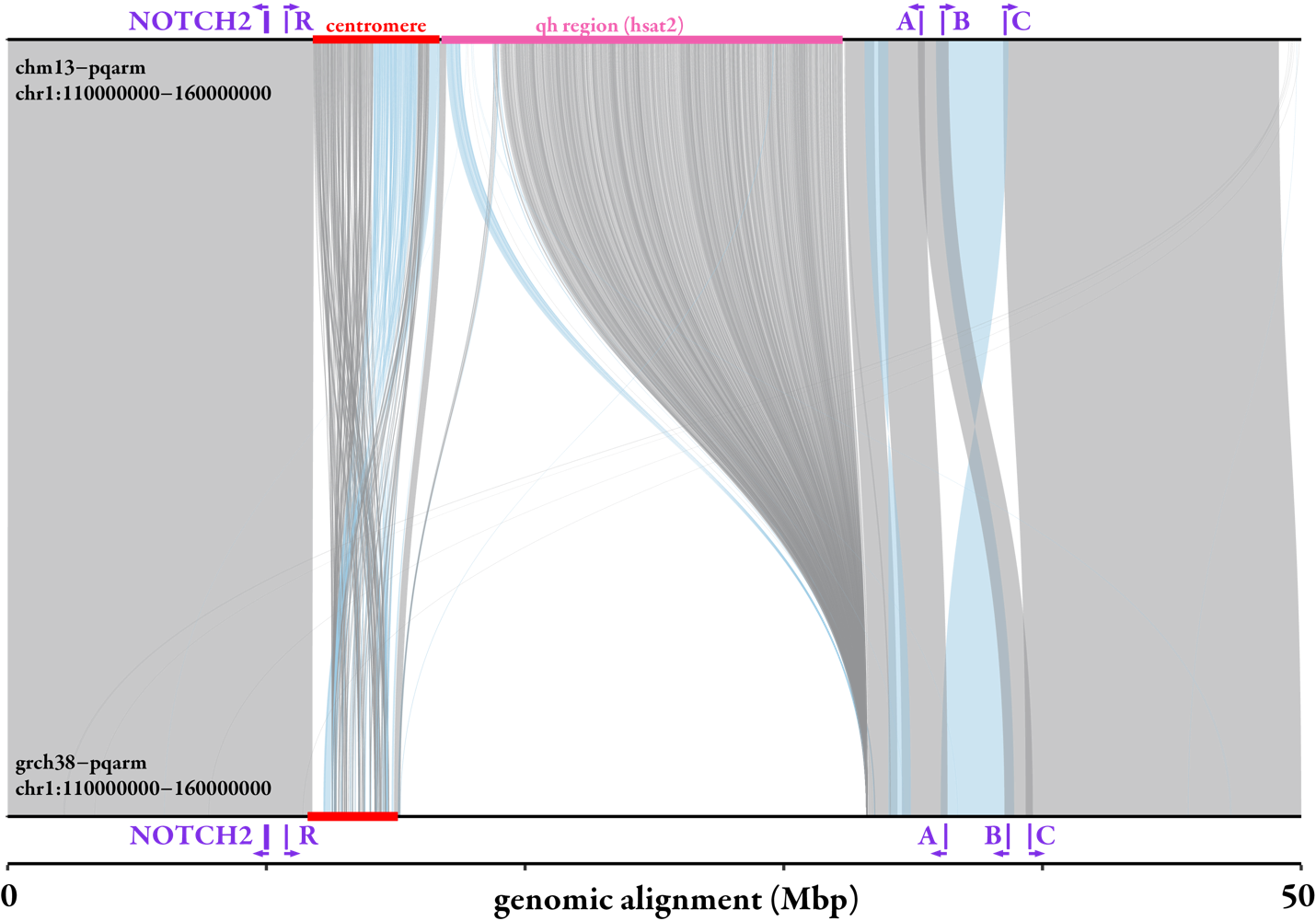


**Supplementary Figure 1. T2T-CHM13 comparison to GRCh38.** Organization of the *NOTCH2/NL* region in the completed T2T haploid assembly CHM13 against the previous reference, GRCh38. The T2T-CHM13 assembly has expanded the sequence and annotations that cover the centromere (red) and qh region (pink), which are two large satellite sequences that separate the two *NOTCH2/NL* (purple) loci. A long inversion around *NOTCH2NLB* that may include both *NOTCH2NLA/C* on either side changes the gene’s orientation between the two assemblies (relevant alignments have increased color opacity).

**
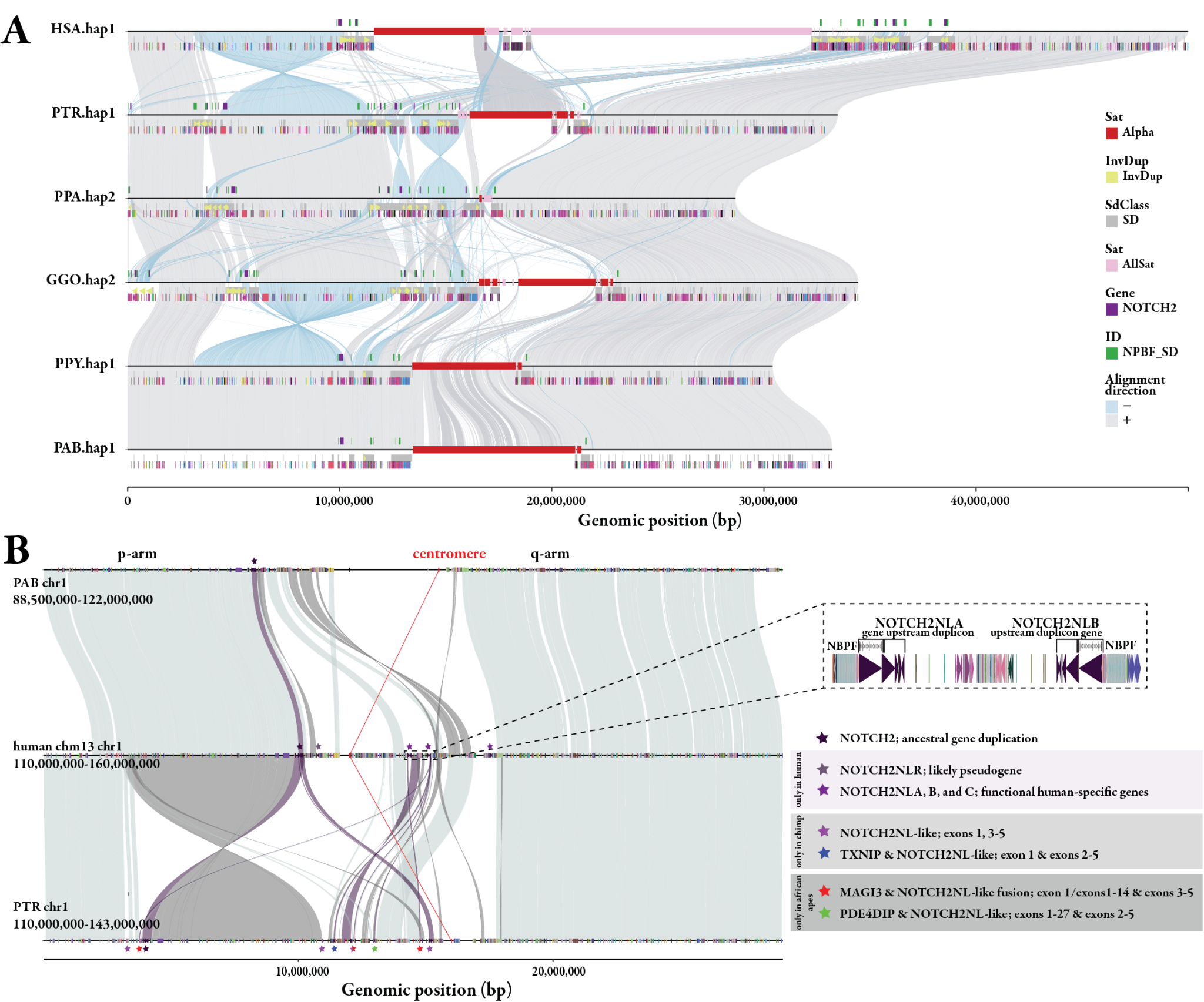
**

**Supplementary Figure 2. NHP and human chromosome 1 evolutionary rearrangements.** **a)** Stacked SVbyEye plot of human chromosome 1 compared to syntenic chromosomes in other apes over 30-50 Mbp of the pericentromeric region. Directly aligned sequence (gray) is compared to inversion rearrangements (light blue) with various annotations including *NOTCH2NL* and *NBPF* genes, satellites, and others. **b)** A zoomed-in comparison including only chimpanzee, orangutan, and human where centromeric sequence has been masked to highlight the non-orthologous location of NHA *NOTCH2NL* transcripts (colored stars) as described by Fiddes et al. (2018).


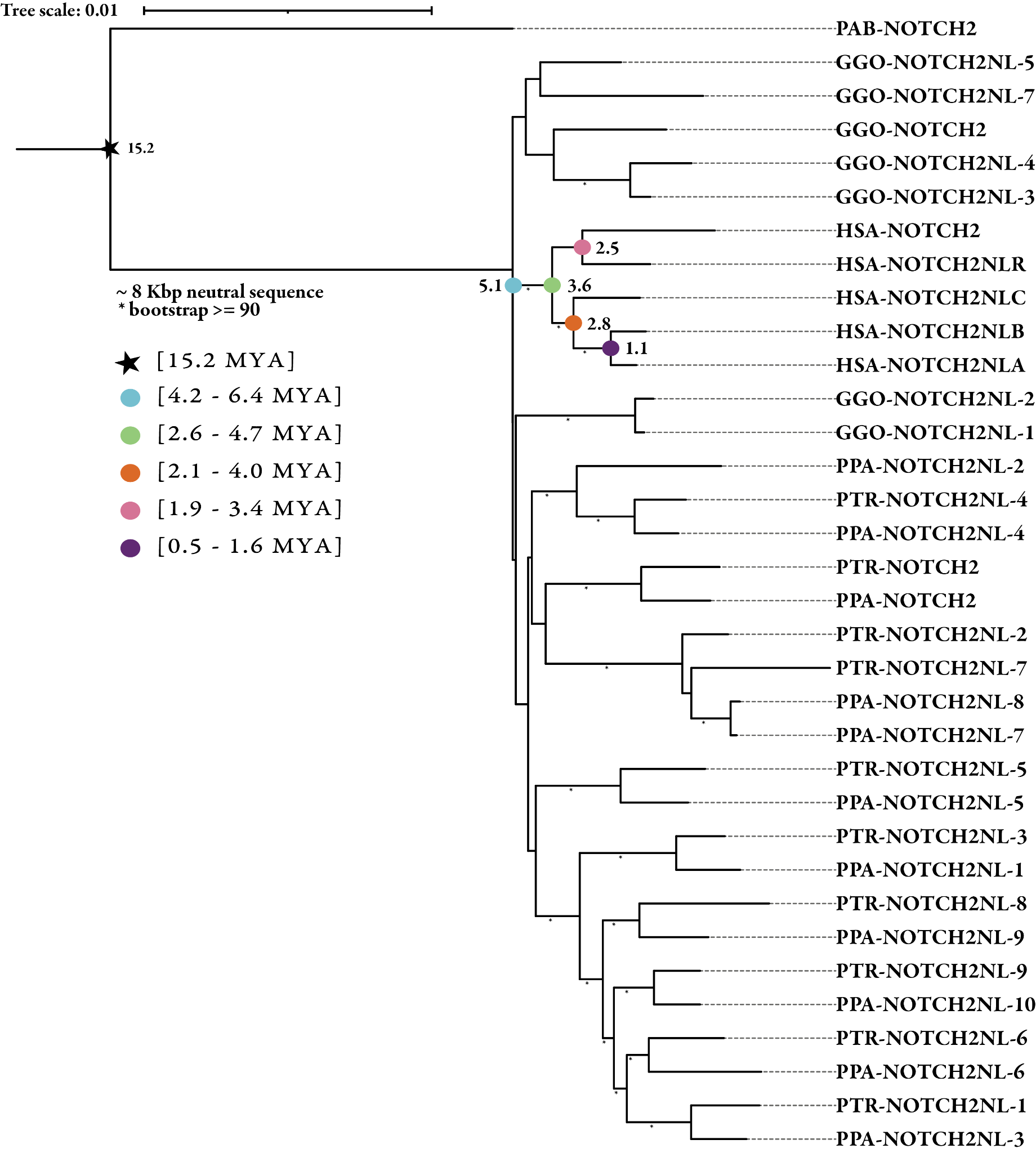


**Supplementary Figure 3. Extended NHA phylogeny.** An alternate maximum likelihood phylogeny based on a multiple sequence alignment of 8 kbp from intron 3 of *NOTCH2/NL* sequence from paralogs of five ape species, using Sumatran orangutan as an outgroup. Boots representing 25/26 NHA homologs. Bootstrap support (>90%) is indicated (asterisk) and is less robust than the phylogeny in Figure 3A but contains more taxa. Estimated divergence times of human paralogs and their confidence intervals are indicated (multicolored dots). Timings were based on human–orangutan divergence time of 15.2 MYA (Methods).

**
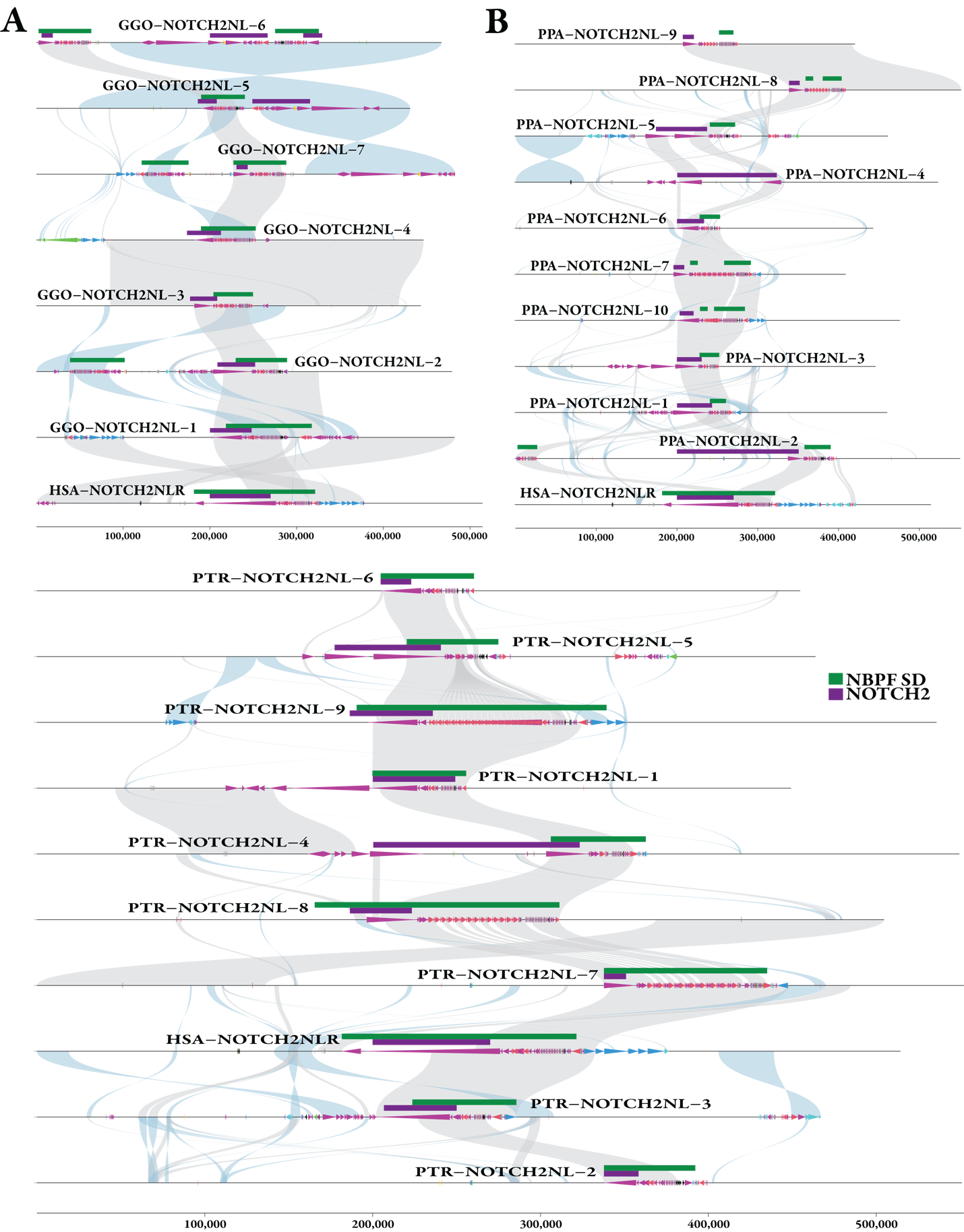
**

**Supplementary Figure 4. Ladder alignment of NHA *NOTCH2NL* homologs.** Gorilla, bonobo, and chimpanzee self-alignments (with human *NOTCH2NLR*) show a consistent association between the *NOTCH2/NL* duplication (purple) and the core duplicon *NBPF* (green). However, the breakpoints of these alignments are largely different not just from humans, but each other.

**
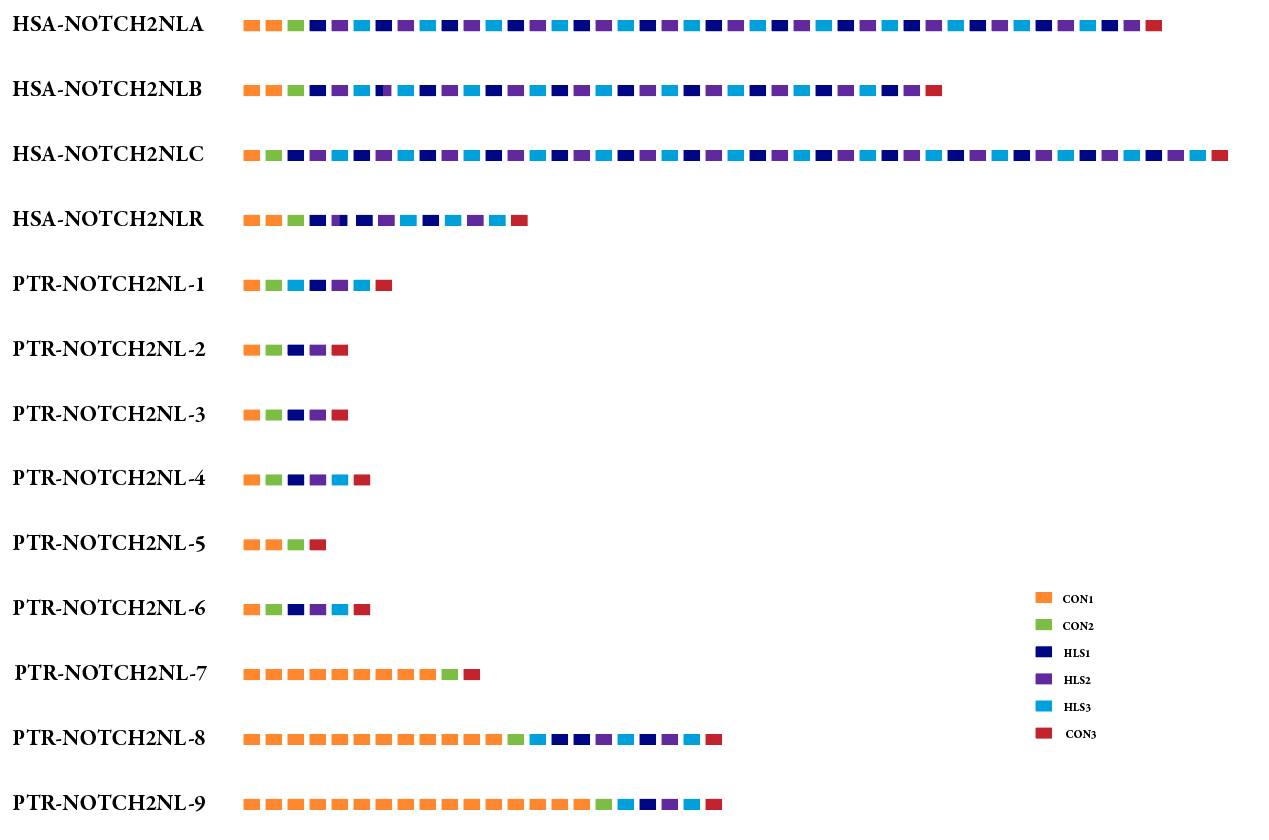
**

**Supplementary Figure 5. NBPF duplicon analysis.** For each of the HSA and PTR *NOTCH2NL* copies, the *NBPF* directly downstream is represented in terms of the Olduvai domains present—CON1, CON2, CON3, HLS1, HLS2, HLS3, based on Fiddes et al. (2019). The *NBPF* copies downstream of *NOTCH2NL* copies in PPA and GGO show similar patterns of Olduvai repeats as well.


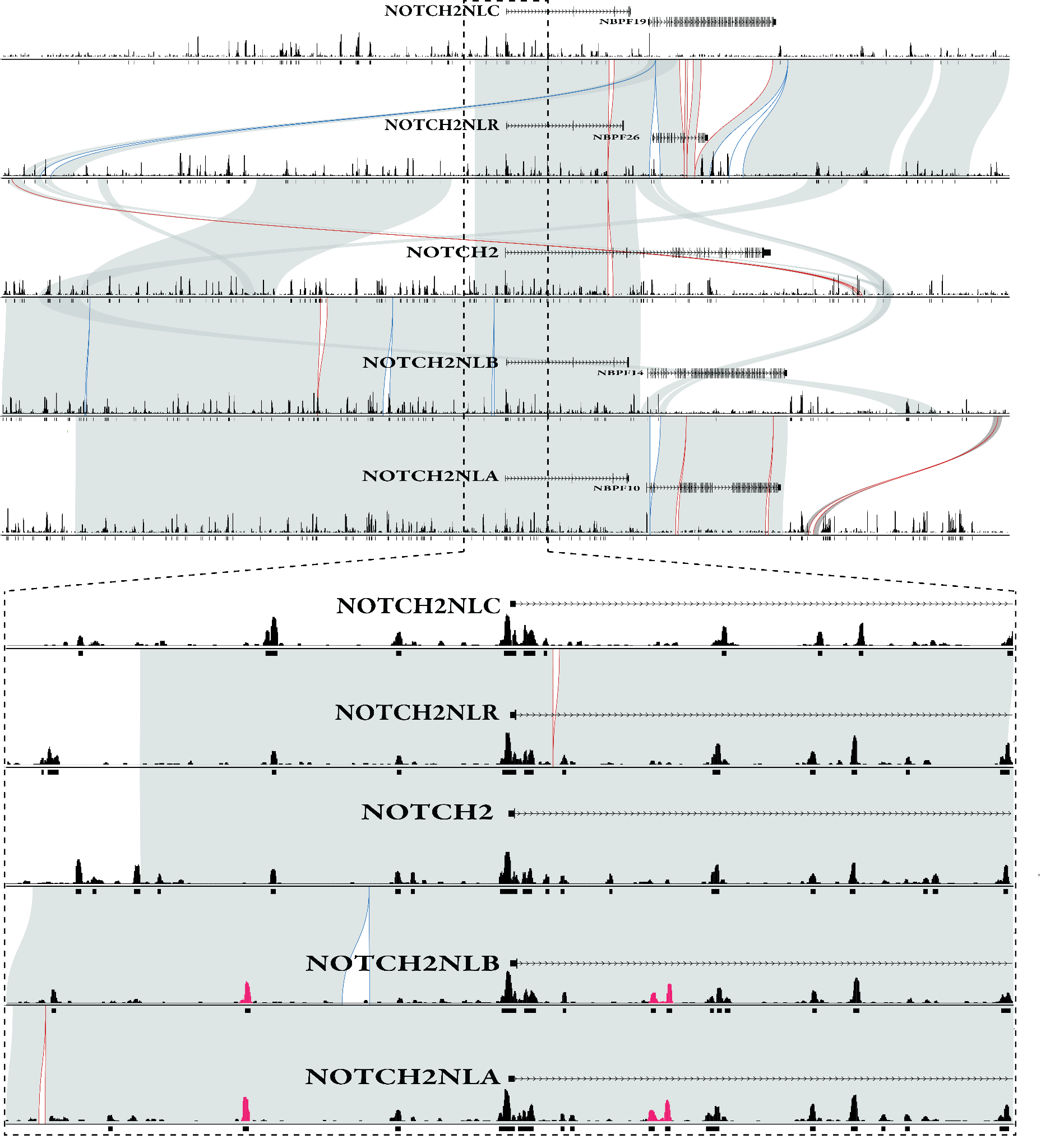


**Supplementary Figure 6. Regulatory architecture of T2T-CHM13.** Fiber-seq chromatin actuation peaks for each T2T-CHM13 *NOTCH2/NL* paralog in the context of homology (gray) and gene model 300 kbp on either side of the TSS. Examples of signals of paralog-specific actuation (pink) are shown in pop-out panel (25 kbp on either side of TSS) in *NOTCH2NLA* and *NOTCH2NLB*. Structural variants like insertions (blue) and deletions (red) are outlined.

**
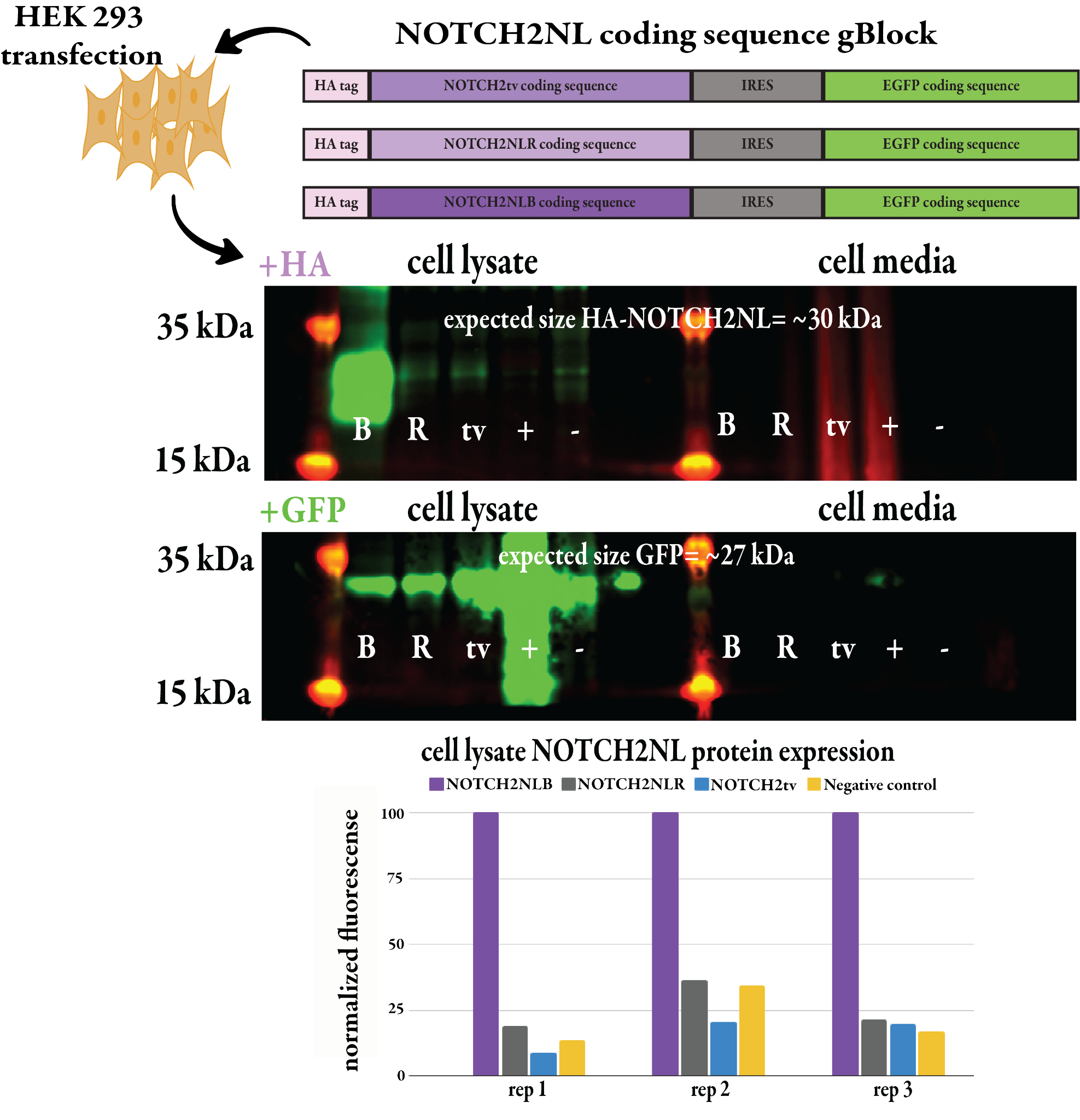
**

**Supplementary Figure 7. NOTCH2tv protein expression.** gBlocks containing an HA antibody tag, *NOTCH2NL* CDS, and *EGFP* CDS were cloned into vector DNA and transfected into HEK293 cells. Antibody staining for HA (NOTCH2NL) and GFP were done on both the cell lysate and media. HA expression fluorescence was normalized using GFP for cell lysate, which shows stable expression of NOTCH2NLB only.
